# Sniffing Fast: Paradoxical Effects on Odor Concentration Discrimination at the Levels of Olfactory Bulb Output and Behavior

**DOI:** 10.1523/ENEURO.0148-18.2018

**Published:** 2018-12-26

**Authors:** Rebecca Jordan, Mihaly Kollo, Andreas T. Schaefer

**Affiliations:** 1 Neurophysiology of Behaviour Laboratory, Francis Crick Institute, London, NW1 1AT, UK; 2 Department of Neuroscience, Physiology & Pharmacology, University College London, London, UK

**Keywords:** Concentration, olfaction, olfactory bulb, oscillations, perception, sniffing

## Abstract

In awake mice, sniffing behavior is subject to complex contextual modulation. It has been hypothesized that variance in inhalation dynamics alters odor concentration profiles in the naris despite a constant environmental concentration. Using whole-cell recordings in the olfactory bulb of awake mice, we directly demonstrate that rapid sniffing mimics the effect of odor concentration increase at the level of both mitral and tufted cell (MTC) firing rate responses and temporal responses. Paradoxically, we find that mice are capable of discriminating fine concentration differences within short timescales despite highly variable sniffing behavior. One way that the olfactory system could differentiate between a change in sniffing and a change in concentration would be to receive information about the inhalation parameters in parallel with information about the odor. We find that the sniff-driven activity of MTCs without odor input is informative of the kind of inhalation that just occurred, allowing rapid detection of a change in inhalation. Thus, a possible reason for sniff modulation of the early olfactory system may be to directly inform downstream centers of nasal flow dynamics, so that an inference can be made about environmental concentration independent of sniff variance.

## Significance Statement

One of the fundamental tasks of the brain is to represent the features of the environment in a stable way. In the olfactory system, it has been hypothesized that changing the way you sniff will alter the concentration of odor coming into the nasal passage, even when the environmental concentration has not changed. Here we show that indeed, the effect of faster sniffing on olfactory bulb responses is very similar to increasing odor concentration. Despite this, mice can easily tell the difference between a change in sniffing and a change in concentration in an olfactory task. To resolve this apparent discrepancy, we suggest and give evidence for ways in which olfactory bulb information about sniffing parameters may be utilized.

## Introduction

For optimal perception, an organism must be able to distinguish between the sensory consequences of its own actions and externally generated stimuli in the environment (Crapse and Sommer, 2008). An example of this comes from controlled eye movements, such as saccades: these act to shift the visual scene on the retina. Such a pattern of motion across the retina could just as easily come from the world moving relative to the eye, and yet we maintain perception of a stable world (von Helmholtz, 1867). An olfactory problem of this nature is the stable encoding of odor intensity—the perceptual correlate of odor concentration (Wojcik and Sirotin, 2014). Increasing concentration is known to affect neural activity in many ways (Mainland et al., 2014). At the level of glomerular input from olfactory sensory neurons (OSNs), increasing concentration enhances the activity of already responsive glomeruli and incorporates new glomeruli into the activity profile, overall resulting in a broadening of the spatial map of activity (Rubin and Katz, 1999; Spors and Grinvald, 2002). Changes in spike rate are also seen at the level of the olfactory bulb (OB) output cells, mitral and tufted cells (MTCs), though this can be a more complex mixture of inhibitory and excitatory effects (Meredith, 1986; Bathellier et al., 2008; Cury and Uchida, 2010; Fukunaga et al., 2012) and is thought to be constrained via inhibitory circuits (Kato et al., 2013; Miyamichi et al., 2013; Fukunaga et al., 2014; Roland et al., 2016). The perhaps more ubiquitous correlates of concentration increase, however, are temporal response changes, notably with early excitation undergoing a latency reduction in OSNs (Rospars et al., 2000; Ghatpande and Reisert, 2011) and MTCs (Cang and Isaacson, 2003; Fukunaga et al., 2012; Sirotin et al., 2015), as well as in the piriform cortex (Bolding and Franks, 2017). This is thought to arise since OSNs will depolarize to threshold more quickly when the concentration profile in the naris is steeper.

In awake mice, sniffing behavior is in continual flux (Welker, 1964; Youngentob et al., 1987; Kepecs et al., 2007; Wesson et al., 2008a, 2009). This might present a problem for concentration coding: changing nasal flow will affect the number of odor molecules entering the nasal passage, altering the concentration profile in the naris despite a stable environmental concentration (Teghtsoonian et al., 1978; Mainland and Sobel, 2006; Shusterman et al., 2018). In other words, altering sniffing may cause self-generated changes in naris odor concentration. Indeed, previous work has shown that faster sniffing can alter firing rates and temporal features of an odor response (Wesson et al., 2009; Carey and Wachowiak, 2011; Shusterman et al., 2011; Cenier et al., 2013; Díaz-Quesada et al., 2018; Jordan et al., 2018). Despite this, previous work suggests that humans can perceive odor intensity independent of the inhalation flow rate (Teghtsoonian et al., 1978), and whether response changes during faster sniffing are similar for a given cell to those evoked by increased concentration is unknown.

Our aims were two-fold: (1) to test the hypothesis that response changes evoked by faster sniffing are the same as those caused by increasing concentration, and (2) to test whether sniff variance would have a negative impact on performance of mice in a fine concentration discrimination task. Using whole-cell patch recordings in awake mice, we show that faster sniffs can indeed evoke both firing rate and temporal response changes identical to those caused by increasing concentration. Surprisingly, however, we show that variance in sniffing has very little impact on the performance of mice during fine concentration discrimination. These results are highly congruent with an accompanying paper using different experimental techniques (Shusterman et al., 2018). Finally, we discuss how the olfactory system could make an inference about whether a response change was caused by concentration change or sniff change, showing that the olfactory bulb encodes sniff dynamics to allow rapid detection of a change in sniffing.

## Materials and Methods

All animal experiments were approved by the local ethics panel of the [Francis Crick Institute]. All mice used were C57BL/6 Jax males aged between 5 and 12 weeks and were obtained by in-house breeding. All chemicals were obtained from Sigma-Aldrich.

### Olfactometry

Odorants were delivered to the animal using a custom-made olfactometer. This consisted of eight different odor channels connecting two manifolds, a clean air channel, and a final dilution channel also carrying clean air. Air was pressure controlled at 1 bar with a pressure regulator (IR 1000, SMC Pneumatics). Flow was computer controlled via mass flow controllers to each manifold such that the olfactometer output provided a constant flow of 2 l/min at all times, meaning that no tactile stimulus accompanied odor pulses. Odor pulses were calibrated to square pulses of different concentrations using a mini photo-ionization detector (miniPID, Aurora Scientific): briefly, pure odor was presented to the PID from an open bottle, and the maximum recorded voltage (V_max_) was assumed to represent 100% saturated vapor pressure. The pulse amplitudes were then calibrated according to this value, such that a given concentration *C* (% saturated vapor pressure) could be specified by attaining a square pulse of amplitude equal to *C* · V_max_/100. Valves and flow controllers were controlled using custom-written LabView software. Odors applied to animals included 2 different odor mixtures (for recordings, either mixture A: methyl salicylate, eugenol, cinnamaldehyde, creosol, and 1-nonanol; or mixture B: guaiacol, valeric acid (+)-carvone, 2-phenyl ethanol, and 4-allylanisol). The components of each mixture were of similar vapor pressure, and proportions were adjusted according to relative vapor pressure values as in a previous study (Jordan et al., 2018). For behavior, either mixture A or pure vanillin odor was applied at various concentrations (Figs. 3 and 4).

### Surgery

Sterile surgical technique was applied during all surgeries. For implantation of the head-plate, mice were anaesthetized with isoflurane in 95% oxygen (5% for induction, 1.5%–3% for maintenance). Local (mepivacaine, 0.5% s.c.) and general analgesics (carprofen 5 mg/kg s.c.) were applied immediately at the onset of surgery. An incision was made dorsally above the cranium overlying the cortex and cerebellum, and periosteal tissue was removed. The surface of the bone was drilled away across the implantation surface using a dental drill, and cyanoacrylate was applied to the sutures between the cranial bones to reduce movement. A stainless steel custom head-plate was then glued to the bone surface with cyanoacrylate, and dental cement was used to reinforce the bond. For mice going on to whole-cell recording, an additional recording chamber was constructed on the bone overlying the right olfactory bulb using dental cement. After surgery, the mice were allowed to recover for 48 h with access to wet diet.

### Whole-cell recordings

On the day of recording, mice were again anaesthetized with isoflurane as above, and carprofen analgesic was injected (5 mg/kg s.c.). A 1-mm-diameter craniotomy was made overlying the right olfactory bulb, and the dura was removed. A layer of 4% low-melting-point agar was then applied to the surface of the bulb, ∼0.5–1 mm thick, to reduce brain movement. Cortex buffer (125 mM NaCl, 5 mM KCl, 10 mM HEPES, 2 mM MgSO_4_, 2 mM CaCl_2_, 10 mM glucose) was used to fill the recording chamber. The animal would then be transferred to the recording rig, head-fixed above a treadmill, and allowed to wake from anesthesia for 20 min. Whole-cell recordings were then made blindly by descending a 5–7-MΩ borosilicate glass micropipette (Hilgenberg, pulled on a DMZ Universal puller, Zeitz Instruments) filled with intracellular solution (130 mM KMeSO_4_, 10 mM HEPES, 7 mM KCl, 2 mM ATP-Na, 2 mM ATP-Mg, 0.5 mM GTP, 0.05 mM EGTA, and in some cases 10 mM biocytin; pH adjusted to 7.4 with KOH, osmolarity = 280 mOsm) through the agar and 180 µm into the olfactory bulb with high pressure. Here pressure was reduced, and the micropipette advanced in steps of 2 µm until a substantial and sudden increase in resistance was observed, indicating proximity to a cell. Pressure was then dropped to zero or below, and a gigaohm seal was attained. Whole-cell configuration was then achieved, and the membrane voltage recording was made in current clamp mode. Identification of mitral and tufted cells was achieved using electrophysiological parameters: an input resistance <150 MΩ, a resting membrane potential between –60 and –40 mV, and an afterhyperpolarization (AHP) waveform conforming to MTC phenotype in an independent component analysis performed as detailed in previous studies (Kollo et al., 2014; Jordan et al., 2018).

**Figure 1.**
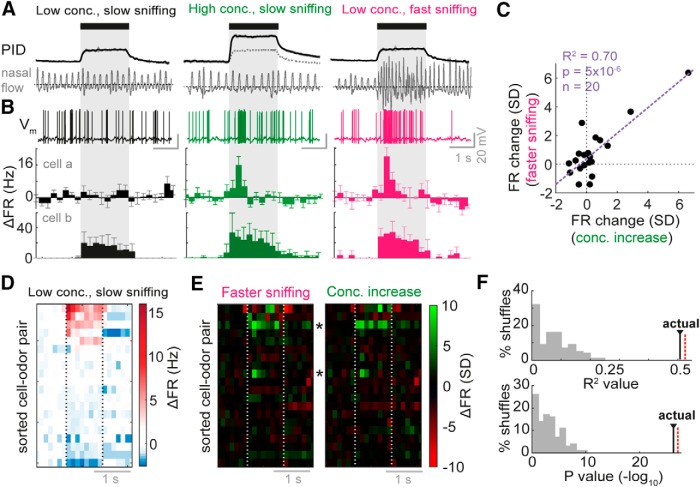
Sniff change and concentration change have very similar effects on FR responses of MTCs. ***A***, Stimulation paradigm during whole-cell recordings. PID traces show response of photoionization detector (magnitude proportional to odor concentration), while nasal flow traces show example sniffing behavior recorded using external flow sensor for the three types of trial. See Figs. 1-1 and 1-2 for details about sniff parameters. Black bar and gray box shows where odor is applied. ***B***, Example odor responses recorded in each stimulus condition. *V_m_* traces show example responses for cell a, while PSTHs below show averaged FR responses in 250 ms time bins for five trials in each case. Bottom-most PSTHs are calculated for a different example, cell b. Error bars show standard deviation (SD). All are aligned to first inhalation onset after odor onset. ***C***, Scatter plot comparing mean FR response change for concentration change and sniff frequency change (normalized by the SD of baseline FR changes in the 2 s before odor stimulus for each cell-odor pair) across first second of odor stimulus. *n* = 20 cell-odor pairs. ***D***, Heatmap of average FR responses for all cell odor pairs in the low concentration, slow sniff frequency condition, ordered by mean FR response. ***E***, Heatmap of FR response differences (difference between PSTHs) normalized by the SD of baseline FR differences in the 2 s before odor stimulus for each cell-odor pair. Concentration increase = high concentration, slow sniffing, minus low concentration, slow sniffing. Faster sniffing = low concentration, fast sniffing, minus low concentration, slow sniffing. Asterisks indicate cell a and cell b examples. ***F***, Top: *R* ^2^ values for correlations across all odor time bins as shown in ***E***, between FR changes due to concentration change and those due to sniff frequency change. Histogram shows *R* ^2^ values for shuffle controls, “actual” shows *R* ^2^ value for real data. Red dotted line indicates value for correlation between FR changes due to concentration increase for two separate sets of high concentration trials. Bottom: as for above, but histogram showing *p*-values for the correlations (–log_10_). See Fig. 1-3 for analysis of membrane potential responses.

**Figure 2.**
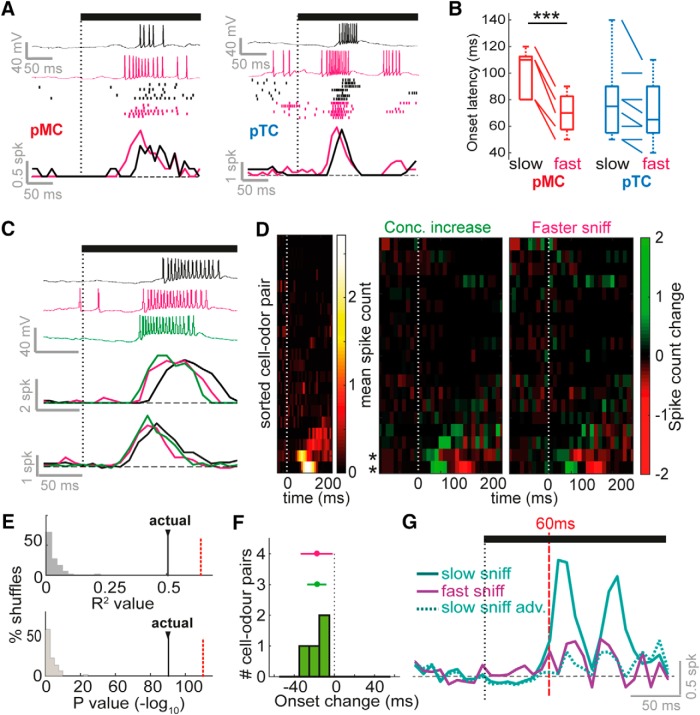
Faster inhalation causes temporal shifts similar to those caused by concentration increase. ***A***, From top to bottom: example *V_m_* traces, spike rasters, and mean spike counts for early excitatory responses for slow inhalation (black) and fast inhalation (pink), for two different cell-odor pairs. The left example is from a putative mitral cell (pMC) and the right example is from a putative tufted cell (pTC). Rasters are ordered (top to bottom) from slowest to fastest inhalation. Black bar and dotted line indicate odor onset aligned to the first inhalation onset. ***B***, Comparison of response onset latencies for excitatory responses evoked by fast and slow sniffs for pMCs and pTCs. See also Fig. 2-1. ***C***, Example *V_m_* traces (above, for one cell) and mean spike counts (below, for two different cells) for early excitatory responses. Black shows response at low concentration evoked by slow inhalation, pink shows response at low concentration evoked by fast inhalation, and green shows response for high concentration evoked by slow inhalation. ***D***, Left: heatmap to show spike counts of all 20 cell-odor pairs in response to low concentration odor stimulus and slow inhalation, for the first 250 ms of stimulation. Cell-odor pairs are sorted by the mean spike count during odor. Middle: heatmap to show difference in spike counts between high concentration and low concentration (evoked by slow inhalation). Left: heatmap to show difference in spike counts between fast inhalation and slow inhalation (low concentration stimulus). ***E***, Top: *R* ^2^ values for correlations across all odor time bins as shown in ***D***, between spike count changes due to concentration increase and due to faster inhalation. Histogram shows values for shuffle controls (see Methods), black bars show value for actual data. Red dotted line indicates value for correlation between spike count changes due to concentration increase for two separate sets of high-concentration trials. Bottom: as for above, but histogram showing *p*-values for the correlations (–log_10_). ***F***, Histogram to show excitatory response onset latency changes due to concentration increase. Error bar in green shows mean and SD of this data, and in pink shows the distribution due to sniff changes (from dataset in panel ***B***) for comparison. ***G***, Euclidean distance between population spike count response vectors for high- versus low-concentration stimuli (where data for both came from slow inhalation trials; “slow sniff,” solid cyan), for high concentration and time-shifted low concentration (“slow sniff adv.,” where excitatory latency changes due to concentration change were undone via time shifting of the data; dotted cyan), and for high concentration and low concentration where low concentration data came from fast inhalation trials (“fast sniff,” solid purple).

Altogether, 14 cells were recorded in passive mice and presented with 2 different odor concentrations, as well as puff stimuli to evoke fast sniffing (Figs. 1 and 2). Some cells were presented with two different odor stimuli (two different mixtures), resulting in 20 cell-odor pairs in total. Concentrations were presented in a pseudorandom order, and puff stimuli occurred on a random subset of trials only for the low concentration. Puff stimuli were applied simultaneously with the odor stimuli with a gentle clean air stream to the flank. For some analyses, such as Figs. 2*A*,*B*, 2-1, 5, 5-1, and 5-2, data were supplemented with previously recorded cells from the passive mouse presented with the same odor mixtures at 1% vapor pressure (*n* = 6 and *n* = 38, respectively).

10.1523/ENEURO.0148-18.2018.f1-1 Extended Figure 1-1 Effect of puff stimulus on sniff behaviour. (A) Example nasal flow traces from one animal during a control trial (no puff stimulus accompanying 2 s odor stimulus) and a trial with a puff stimulus. Shaded area shows odor stimulus. Black ticks indicate inhalation onsets. (B) Plots to show average change in sniff frequency, mean inhalation duration, and first inhalation duration between five control (black) and five puff (orange) trials for all 20 cell-odor pairs. Download Extended Figure 1-1, TIF file.

10.1523/ENEURO.0148-18.2018.f1-2 Extended Figure 1-2 Relationships between different sniffing parameters. (A) Example nasal flow trace during an inter-trial interval (no odor), with sniffs colored according to their inhalation duration (blue to red = long to short duration). Black ticks show time of inhalation onset, orange plot shows peak inhalation slope for each inhalation aligned to the inhalation onset, and green plot shows inhalation duration for each inhalation. (B) Example correlation between inhalation duration and peak inhalation slope for 1988 sniffs in 1 animal (top), and histogram of correlation R values between inhalation duration and peak inhalation slope across 50 animals. Black bars indicate significant correlations. (C) As for B, but for correlation between sniff duration and inhalation duration. As expected from constraints on duty cycle during respiration, we found a biphasic relationship between sniff duration and inhalation duration, with a linear correlation for sniffs < 250 ms duration (magenta) and a plateau for sniffs > 250 ms duration (blue). This resulted in high R values for sniffs < 250 ms duration and low R values for sniffs > 250 ms duration. (D) As for B, but for the correlation between the previous sniff duration and the current inhalation duration. Download Extended Figure 1-2, TIF file.

10.1523/ENEURO.0148-18.2018.f1-3 Extended Figure 1-3 Changes in subthreshold response are more inhibitory for concentration increase than for fast sniffing. (A) Example average subthreshold response traces for low concentration, slow sniffing (black), high concentration, slow sniffing (green) and low concentration, fast sniffing (magenta), for two different cells, cell c (top) and cell d (bottom). Each trace is the average of 5 spike-subtracted trials. (B) Scatter plot to show average change in membrane potential response for the first 1 s of the odor stimulus for concentration increase (high conc.-low conc.) and sniff frequency change (fast sniffing-slow sniffing). (C) Cumulative histograms of membrane potential response change for concentration increase (green) and sniff frequency increase (magenta). P = 0.03, paired t-test. Download Extended Figure 1-3, TIF file.

10.1523/ENEURO.0148-18.2018.f2-1 Extended Figure 2-1 Additional data for sniff-induced temporal shifts in odor response. (A) Heatmaps of mean spike count for 13 cell-odor pairs showing early excitation in response to the odor presented, for both slow inhalation (top) and fast inhalation (middle). White dashed line indicates odor onset aligned to the first inhalation onset. Cell-odor pairs are sorted from short to long response onset latency (during slow inhalation). Bottom heatmap shows the difference between the two above (fast-slow). White solid and dotted line indicates onset latency of each cell-odor pair for slow inhalation. Blue line indicates onset latency for fast inhalation. (B) Histogram of onset latency changes (fast-slow) for all 13 cell-odor pairs. Errorbar shows mean and SD. (C) Scatter plot to show relationship between onset latency for slow inhalation, and the onset change between fast and slow inhalation (ΔOnset). (D) Correlation between response onset latency and peak spike count (analysed within 10 ms time bins) for early excitatory odor responses evoked by a slow sniff. Blue data comes from pTCs and red data comes from pMCs. Boxplots compare the two parameters for pTCs and pMCs. (E) Comparison of response onset latency change (fast-slow sniff) for pMCs and pTCs. (F) Above and below plots are for two different example cells. Left: plot to show first inhalation duration during odor stimulation sorted from shortest to longest for all trials for one cell. Right: heatmap of spike count for the cell during odor stimulation for trials sorted by first inhalation duration as in left plot. White dotted line indicates where odor is on (aligned to first inhalation onset). Download Extended Figure 2-1, TIF file.

### Behavioral task and training

On day 0 (48 h after surgery), mice with head-plates implanted would begin water restriction. On day 1, mice were habituated to the experimenter and hand-fed 0.5 ml of highly diluted sweetened condensed milk with a Pasteur pipette. On day 2, mice were habituated to head-fixation: mice were head-fixed above a treadmill and allowed access to free reward on licking the reward port (licks were detected using an IR beam). On day 3, successfully habituated mice underwent operant conditioning with repeated presentations of CS+ concentration of the odor mixture until the mouse learned to lick in the 1 s after odor offset to receive the reward. On day 5, the CS– concentration was also presented alongside the CS+ concentration in a pseudorandom order, until the mice learned to refrain from licking to the CS–. Licking to the CS– would evoke an addition of 6 s to the intertrial interval. Five mice were trained with high-concentration stimuli as the CS+ (“high go”), and three mice were trained on the reverse contingency (“low go”). On days 6–8, mice would be presented with five different concentrations (three additional concentrations spanning the range between the previously two learned concentrations), and contingencies as depicted in Fig. 4*A*. On day 9, five mice went on to a final session: after observing criterion performance on the binary odor concentration task with the mixture as learned previously, the odor would switch to vanillin with the same contingency between concentrations.

Mice were carefully monitored to maintain their body weights >80% of their prerestriction weight and were ensured a minimum of 1 ml water per day regardless of performance. Any mouse exceeding this weight loss or showing signs of distress was immediately returned to water access.

### Sniff measurement

Nasal flow was recorded by placing a flow sensor (FBAM200DU, Sensortechnics) externally in close proximity to the nostril contralateral to the side of whole-cell recording and sampled at 1 kHz. The position of the sensor was manually optimized at the start of each session such that all sniff cycles were captured with a high signal-to-noise ratio.

### Data analysis

In all cases, 5%–95% confidence intervals were used to determine significance unless otherwise stated. In all figures, a single asterisk denotes *p* < 0.05, a double asterisk denotes *p* < 0.01, and a triple asterisk denotes *p* < 0.001. Means and error bars showing a single standard deviation either side are used in all cases where comparing normally distributed data of equal variance. Lilliefors tests were used to determine if a dataset was normally distributed. In the case of normal distributions, two-sided Student’s *t* tests were used for comparison of means and Bartlett tests used to compare variances, unless otherwise stated. If data were not normally distributed, or where two datasets were not of equal variance, ranksum tests were used to compare the medians, and Browne–Forsythe tests used to compare variance, unless otherwise stated. Boxplots are used to represent such data (data comparisons of unequal variance, or non–normally distributed data), where median is plotted as a line within a box formed from 25th (q1) and 75th (q3) percentile. Points are drawn as outliers if they are larger than q3 + 1.5 × (q3 – q1) or smaller than q1 – 1.5 × (q3 – q1). Superscript letters listed with *p*-values correspond to the statistical tests shown in Table 1.

**Table 1.** Statistical analysis

Location	Data structure	Statistical test	95% confidence intervals
a	Paired response onset latencies (fast vs slow sniffs), *n* = 13 cells	Paired *t* test	–25 to –7 ms
b	Paired response onset latencies (fast vs slow sniffs), *n* = 5 pMCs	Paired *t* test	–39 to –22 ms
c	Paired response onset latencies (fast vs slow sniffs), *n* = 8 pTCs	Paired *t* test	–16 to 1 ms
d	Normal distributions of equal variance	Unpaired Student’s *t* test, 2-tailed	11 < 23 < 34 ms
e	Paired response onset latencies (high vs low concentration, *n* = 4)	Paired *t* test	2.3 to 33 ms
f	SD in inhalation duration for passive (*n* = 23) and concentration go/no go mice (*n* = 7), calculated for each block (1 block = 10 trials)	Two-way ANOVA on SD in inhalation duration [factors: block #, behavior (passive vs concentration go/no go)]	Multiple comparison test: –10 < 5 < –2 ms
g	Go rate for fast and slow sniff trials for each concentration (5), for *n* = 3 mice trained on low Go contingency	Three-way ANOVA on go rates [factors: mouse, concentration, sniffing (fast vs slow)]	Multiple comparison test: –20 < –10 < –1%
h	Go rate for fast and slow sniff trials for each concentration (5), for *n* = 4 mice trained on high Go contingency	Three-way ANOVA on go rates (factors: mouse, concentration, sniffing (fast vs slow))	Multiple comparison test: –22 < –15 < –7%
i	Difference in go rate between fast and slow sniff trials for each concentration (5), for mice trained on two different contingencies: “low go (*n* = 3 mice)” and “high go (*n* = 2 mice)”	Two-way ANOVA on differences in go rate (factors: contingency, concentration)	Multiple comparison test: –16 < –4 < 7%
j	Normal distributions of equal variance	Paired t-test	–15 to 4%
k	Go rate for probe trials and control trials for each concentration (5), for *n* = 3 mice trained on low Go contingency	Three-way ANOVA on go rates [factors: mouse, concentration, trial type (probe vs control)]	Multiple comparison test: –16 < –7 < 3%
l	Go rate for probe trials and control trials for each concentration (5), for *n* = 4 mice trained on high Go contingency	Three-way ANOVA on go rates [factors: mouse, concentration, trial type (probe vs control)]	multiple comparison test: –19 < –8 < 3%
m	Difference in go rate between probe and control trials for each concentration (5), for mice trained on two different contingencies: “low go (*n* = 3 mice)” and “high Go (*n* = 2 mice)”	Two-way ANOVA on differences in go rate (factors: contingency, concentration)	Multiple comparison test: 13 < 1 < 16 %
n	Paired reaction time data (fast vs slow sniffing, *n* = 5 mice)	Paired *t* test	0.0 to 70 ms
o	Paired reaction time data (puff vs control, *n* = 5 mice)	Paired *t* test	–61 to 50 ms
p	Contingency table (significant vs non-significant *R* ^2^, actual data vs shuffle controls)	Fisher’s exact test	3.4 to 18.3
q	Contingency table (significant vs non-significant *R* ^2^, actual data vs shuffle controls)	Fisher’s exact test	3.5 to 23.3
r	Contingency table (significant vs non-significant *R* ^2^, actual data vs shuffle controls)	Fisher’s exact test	5.9 to 33.3

### Sniff parameters

Using the recording of nasal flow, different sniff parameters could be extracted. First, inhalation peaks were detected using Spike2 algorithms that mark each peak above a certain threshold voltage manually defined by the user, such that all inhalations were included and no false positives were present. Inhalation onset was defined as the nearest time point before inhalation peak at which the flow trace reached zero. Inhalation offset was similarly calculated as the first time point after inhalation peak where the flow trace reached zero. Inhalation duration was defined as the difference in time between inhalation onset and offset. Peak inhalation slopes were calculated by detecting the peak value of the differentiated flow waveform 50–0 ms before inhalation peak. Sniff duration was calculated as the time between subsequent inhalation onsets. Sniff frequency was calculated by taking the inverse of the mean sniff duration within the odor time period.

### Spike rate responses and onsets

Note that when comparing response changes due to concentration and response changes due to sniff change, the same number of trials was used in both conditions.

Long timescale (Fig. 1): For each cell, mean spike count was calculated in 250-ms time bins for the full 2-s odor stimulus. These were then averaged across trials to generate PSTHs for low concentration and fast sniffing (five trials of lowest mean inhalation duration), low concentration and slow sniffing (five trials with highest mean inhalation duration), and high concentration and slow sniffing (five trials of highest mean inhalation duration). Values were quadrupled to estimate fining rate (FR) in Hz.

Short timescale (Fig. 2): For each cell, spike counts were calculated in 10-ms time bins for only the first 250 ms from odor onset (aligned to first inhalation after odor onset). These spike counts were then averaged across trials for low concentration and fast inhalation (>70^th^ percentile peak inhalation slopes), low concentration and slow inhalation (<30^th^ percentile inhalation slopes), and high concentration and slow inhalation (<30^th^ percentil peak inhalation slopes). Onset for excitatory responses was defined at the point at which the mean spike count exceeded the mean +2 standard deviations (SDs) of the baseline spike rate in the 250 ms before odor onset, and remained there for at least 2 consecutive points.

### V_m_ responses

To analyze subthreshold responses in absence of spiking activity, spikes and their AHPs were subtracted from the trace. This was done by first using the “wavemark” tool in Spike2 to detect spikes by thresholding and matching them to a generated spike waveform template. The length of this spike waveform template was manually adjusted for each cell according to its AHP length, but was usually around –4 ms to 20–30 ms relative to spike peak. A trace was then generated containing all detected spike waveforms connected by zero values, and this was subtracted from the original voltage trace.

### Correlations between response changes due to sniffing and concentration change

For both long- and short-timescale mean FR responses, changes in FR response were calculated for sniff change (Δ*S*, fast minus slow sniffing, low-concentration odor) and concentration change (Δ*C*, high minus low concentration, slow sniffing). For all cell-odor pairs across the sample, a single regression was made between FR changes for sniff change and FR changes for concentration change in the corresponding time bins, generating an actual *R* ^2^ and *p* value (Figs. 1*F* and 2*E*). For shuffle controls, low-concentration trials were shuffled with respect to the sniff behavior on each trial, and the same analysis was repeated 100 times. To compare how strong the correlations were in a relevant way, the high-concentration trials were randomly separated into two halves, and a linear regression was made between the changes in FR for each half (relative to low-concentration trials) as above. This allowed us to compare *R* ^2^ values for correlations between FR changes due to concentration increase versus concentration increase (Δ*C*_1_ versus Δ*C*_2_, different trial subsets) and FR changes due to concentration increase versus faster sniffing (Δ*C* versus Δ*S*).

### Euclidean distance analysis of concentration discriminability

In reference to Fig. 2*G*, Euclidean distance was taken across the population between mean spike counts for high concentration and low concentration (slow inhalation). This generated a measure of discriminability between concentrations when the inhalation was slow for both concentrations. To test how much of the discriminability was due to latency shift of excitation between low and high concentrations, responses that underwent a detectable latency shift had their spike count response to low concentration manually shifted earlier according to the latency shift occurring between high and low concentration. Euclidean distance was then recalculated between spike counts for high concentration and the latency-shifted spike counts at low concentration. Finally, Euclidean distances were calculated between spike counts for high concentration (slow inhalation) and low concentration (fast inhalation). Time for discrimination was calculated, if possible, as the point at which the Euclidean distance exceeded the mean + 2 SDs of the baseline Euclidean distance (250 ms before odor onset) for at least 2 consecutive 10-ms time bins.

### Baseline activity correlations with inhalation duration

For each cell (*n* = 45), 1000–2000 sniffs were analyzed in absence of odor. Sniffs were categorized according to their inhalation duration, 35–45, 45–55, 55–65 ms and so forth. For each individual sniff, different parameters were calculated from the corresponding neural activity. Mean membrane potential was calculated from the subthreshold membrane potential occurring from 0 to 250 ms from inhalation onset. Peak membrane potential was designated as the maximum membrane potential within 30–250 ms after inhalation onset, and time of the peak membrane potential was determined as the time of this maximum membrane potential relative to inhalation onset. Spike counts were calculated by summing all action potentials occurring within the same time frame. To calculate the correlations for each parameter, each was averaged across all sniffs within the category, and regression analysis was used to generate an *R* and *p* value between the resulting average parameters and the corresponding inhalation duration (minimum of the category). For each cell, inhalation duration categories were excluded from the correlation if they contained <25 sniffs, and cells that had <5 valid categories were additionally excluded. For shuffle controls, inhalation duration was shuffled throughout the data, and the regression analysis was repeated 10 times per cell.

### Euclidean distance analysis of detectability of sniff change

For this analysis, only cells with >50 sniffs during baseline in each category, 55–65, 75–85, and 95–105 ms inhalation duration, were included. A random subset of 25 sniffs in each group were selected, and spike activity within these samples were used to construct PSTHs. Each PSTH was normalized such that the first 30 ms started at zero Hz on average. PSTHs were put in sequence, either 3 consecutive 95-ms inhalation duration sniffs (control sequence), or the same sequence but with the final sniff of a different inhalation duration, either 75 or 55 ms. Euclidean distance across the population of these sequences were then calculated between the control sequence and sequences ending in 55- or 75-ms inhalation duration sniffs. Detection time for the change in inhalation duration was calculated where the Euclidean distance in the last sniff exceeded the mean + 2 SDs of the baseline Euclidean distance from the first 2 sniffs.

### Phase preference and putative mitral and tufted cell boundaries

The sniff-V_m_ modulation properties of each cell were calculated from the intertrial intervals (i.e., in absence of odor) as in previous studies (Fukunaga et al., 2012; Jordan et al., 2018). Due to the high variability of sniff behavior in awake mice, analysis was restricted to sniff cycles between 0.25 and 0.3 s in duration, where the preceding sniff cycle was also within this range. Mean *V_m_* from the spike-subtracted *V_m_* trace was taken as a function of sniff cycle phase for at least 150 sniffs, and this was plotted as Cartesian coordinates. The angle of the mean vector calculated by averaging these Cartesian coordinates was taken as the phase preference of the cell. To determine putative mitral cell (MC) or tufted cell (TC) type based on phase preference, we used the phase boundaries determined previously (Jordan et al., 2018): pMCs were defined as cells with phase preferences within the phase boundaries 0.39–4.11 radians (inhalation), and pTCs were defined as those with phase preferences within the remaining boundaries (exhalation).

### Prediction of inhalation duration with peak spike rates with simple linear model

Sniff cycles occurring in absence of odor (during the intertrial intervals) from 25 whole-cell recorded neurons (19 pMC, 6 pTC) were divided into 10-ms bins according to inhalation duration (e.g., 30–40, 40–50 ms, and so forth) and used to construct pseudo-population activity for individual sniff cycles of a given inhalation duration. Only cells with at least 20 sniff cycles for each inhalation duration bin were included. From each cell, the peak spike rate (smallest interspike interval) within 400 ms of sniff onset was calculated for each sniff cycle. The peak spike rates across the pseudo-population for 13 random sniff cycles within each inhalation duration category were used to generate a simple linear model to predict the inhalation duration. The resulting model was then tested on the remaining 7 sniff cycles, and the relationship between predicted and true inhalation duration was compared (Fig. 5*E*).

### Modulation of sniff-activity relationships across phase preference

In reference to extended data Fig. 5-1, to determine if the sign of relationship between inhalation duration and the various activity parameters was related to the sniff phase preference of the cell, *R* values for the various correlations were plotted as a function of phase preference. Only correlations with a significant *p* value (<0.05) and an *R* ^2^ > 0.6 were included. A sliding window of 2 radians was then used to calculate the mean *R* value for all cells with phase preference within the window, resulting in a mean *R* value as a function of phase preference. The modulation strength of mean *R* value as a function of phase was then calculated: the plot of mean *R* value was normalized to the minimum value across all phases, and the result was plotted as Cartesian coordinates. The length of the mean vector calculated by averaging these Cartesian coordinates was taken as the modulation strength of the *R* value across phase space. To determine the significance of this modulation, *R* values were shuffled with respect to phase preference 10,000 times, and the resulting distribution of shuffled modulation strength was compared to the value for the unshuffled data.

### Learning time and reaction time

**Figure 3.**
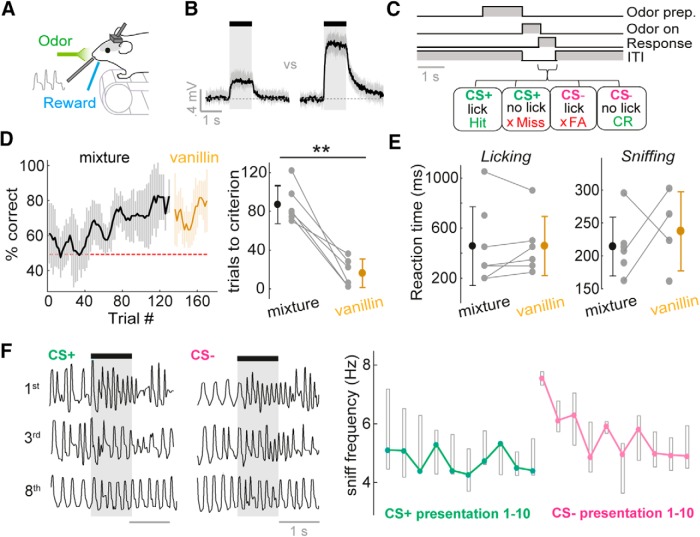
Mice rapidly learn to discriminate concentrations on fast timescales. ***A***, Diagram of head-fixed behavior setup. ***B***, Average PID traces for concentration go/no-go stimuli. Shaded area shows SD. See Fig. 3-1*A* for odor outlet flow traces. ***C***, Concentration go/no-go task sequence. See Fig. 3-1*B* for training protocol. ***D***, Left: average learning curve for eight mice. Percentage correct is calculated as a moving average over 5 CS+ and 5 CS– trials. Shaded area indicates SD. Mice are initially trained on two concentrations of an odor mixture, and subsequently tested on the same two concentrations of vanillin. Right: distribution of learning times to criterion (four successive learning curve points at or above 80% correct), for the odor mixture and vanillin. ***E***, Left: distribution of reaction times (RTs) calculated from licking behavior for the odor mixture and vanillin. Right: as for left, but for RTs calculated from sniffing behavior (see Methods). ***F***, Left: example sniff traces for the 1st, 3rd, and 8th presentation of the CS+ and CS– concentrations for the initial concentration discrimination learning session. Note that in this session, the CS+ concentration is first presented 10 times to ensure retention of the lick pattern learned the day before, and then the CS– is interleaved in a pseudorandom order. Right: plot to show median sniff frequency across 8 mice (regardless of concentration-reward contingency) for presentations 1–10 of the CS+ and CS– concentration in the first 2 concentration discrimination sessions. Boxes show upper and lower quartiles.

For the generation of learning curves as in Fig. 3, a moving window was used across five consecutive CS+ and five consecutive CS– trials and advanced by one trial on each step, and a percentage correct was calculated. The trial at which this reached at least 80% correct for five consecutive points was deemed the learning time.

**Figure 4.**
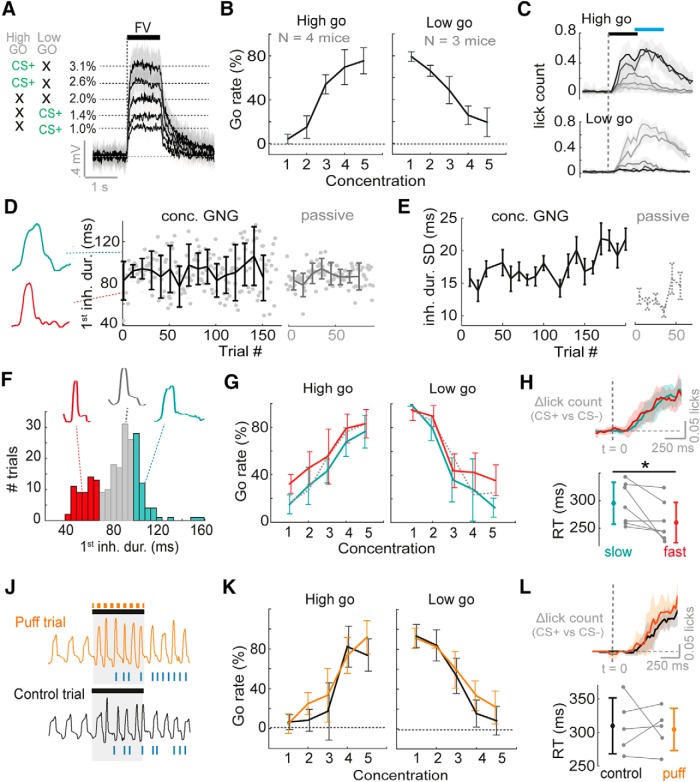
Variance in inhalation has no overt impact on concentration discrimination performance. ***A***, Diagram to show average PID traces for the five different concentrations and contingency schemes. Shaded area shows SD. To the left the contingencies are shown for “high-go” and “low-go” trained mice, with black crosses indicating CS– stimuli. ***B***, Average go rate (percentage of trials with a go response) across mice for all five concentrations. ***C***, Mean lick counts averaged across mice for the five different concentrations (darkest = strongest) for both “high go” and “low go” training contingencies. Black bar indicates odor stimulus, and blue bar indicates response period. ***D***, Plot to show inhalation duration for first inhalation of the odor stimulus across trials, for the first session of one example mouse performing the five-concentration go/no-go task (“concentration GNG”), and for a passively exposed mouse (“passive”). Error bars show SD for each 10-trial block. Example representative nasal flow waveforms for single sniffs are shown to the left. ***E***, Mean SD for the first inhalation duration (ms) during the odor stimulus, for seven mice performing five-concentration go/no-go in their first session, and for passively exposed mice (*n* = 23). SD is calculated for each 10-trial block of a session for each mouse. Error bars show standard error. ***F***, Example histogram of inhalation durations of the first sniff during an odor stimulus across trials for one mouse. Data for each mouse is partitioned into fast inhalations (<30^th^ percentile, red), slow inhalations (>70^th^ percentile, cyan), and other (gray). Example representative nasal flow waveforms for a single sniff of each subset are shown. ***G***, Go rate as a function of concentration when splitting trials according to duration of first inhalation as in ***F***. Dotted line shows mean go rate for sniffs with inhalation between 30^th^ and 70^th^ percentile. ***H***, Top: average difference in lick-histograms between CS+ and CS– (highest versus lowest concentration) averaged across all seven mice for slow sniff trials (cyan data) and fast sniff trials (red data) partitioned as in ***F***. Shaded area indicates SD. Dashed line indicates odor stimulus onset aligned to the first inhalation. Bottom plot shows difference in reaction times as measured by licking for fast and slow sniff trials for all seven mice. See also Fig. 3-1*D*. ***J***, Example sniff traces for one animal for a puff trial (a trial in which an air puff to the flank was used to evoke fast sniffing) and an adjacent control trial of the same concentration. Blue ticks indicate licks. ***K***, Mean go rate as a function of concentration across mice for puff trials (orange) versus control trials (black). ***L***, As for ***H***, but now comparing lick distributions and reaction times between puff trials and control trials. See also Fig. 3-1*E*.

10.1523/ENEURO.0148-18.2018.f3-1 Extended Figure 3-1 Additional behavioral data. (A) Mean flow change recorded 1 mm from olfactometer output for high concentration stimulus (red) and low concentration stimulus (blue). Average of 10 trials; shaded area shows standard deviation. Y scale bar is compared to that of nasal flow traces recorded in the same manner to demonstrate the negligible nature of flow change from the olfactometer. (B) Diagram to show training sequence for mice (described in methods). (C) Comparison of changes in the first inhalation duration between physiological and behavioural experiments. Black ('phys.') shows distribution of mean change used for analysis of odor responses for 20 cell-odor pairs recorded in passive mice (as in Figure 2). Purple ('F vs S') shows mean difference between red and cyan sections of the inhalation distribution as in Figure 4F for all mice and concentrations (n = 7 mice x 5 concentrations). Orange 'puff' shows average changes in mean first inhalation duration for puff vs control trials during behaviour (n = 7 mice x 5 concentrations, as in Figure 4J). (D) Average difference in lick-histograms between CS+ and CS- (concentration 4, 2.6%, vs concentration 2, 1.4%) averaged across all 7 mice for slow sniff trials (cyan data) and fast sniff trials (red data) partitioned as in Figure 4F. Dotted line indicates onset of odor stimulus (aligned to the first sniff onset). Right plot shows difference in reaction times as measured by licking for fast and slow sniff trials for all 7 mice. Mean difference in RT (fast-slow) = -92 ± 235 ms, p = 0.34 paired t-test. (E) As for panel D, but now comparing lick distributions and reaction times between puff trials (orange) and control trials (black), as in Figure 4L. Mean difference in RT (puff-control) = -36 ± 125 ms, p = 0.61, paired t-test. Download Extended Figure 3-1, TIF file.

Reaction time calculations were based on 10 or more trials of at least 80% correct performance. From lick behavior, for each trial, lick probability was calculated in a moving time window of 100 ms, aligned to the first inhalation onset after final valve opening. The difference between the probability of licking for CS+ and CS– stimuli for each time window was calculated, and the leading edge of the first window at which this calculated difference significantly deviated (>2 SD) from the values calculated in the 2-s window before odor onset was considered the reaction time. From sniff behavior, inhalation and exhalation duration values were calculated for each trial as a function of sniff number from odor onset. These values were compared between those calculated for CS+ and CS– using a *t* test, and the reaction time was calculated based on the first inhalation or exhalation within the series to show a significant difference (*p* < 0.05).

## Results

### Changes in sniffing can mimic the effect of increased concentration on firing rate response

We first wanted to determine if the effect of sniff changes on MTC odor response could qualitatively mimic concentration changes at the level of FR change. To do this, we used whole-cell recordings from identified MTCs in awake passive mice, as this allows unbiased sampling from the MTC population in terms of baseline FR, and reliable identification of cell type based on electrophysiology (Margrie et al., 2002; Kollo et al., 2014). On each trial, mice were presented randomly with 2-s-long odor stimuli calibrated to either 1% (low concentration) or 2.5% (high concentration) square pulses. On a small percentage of low-concentration trials, mice also received a gentle air puff to the flank, evoking fast sniffing behavior characterized by high-frequency sniffs and short inhalation durations (Figs. 1*A* and 1-1). For all analyses in the manuscript, “odor onset” (*t* = 0) is defined as the first inhalation onset during the odor stimulus. Note that several parameters of sniffing covary with inhalation duration, including the sniff duration, the previous sniff duration, and the slope of the inhalation (Fig. 1-2). Thus wherever we refer to fast or slow sniffing, this will necessarily refer to differences in these multiple parameters.

During recordings, it was apparent that some cells displayed overt changes in FR with the increase in concentration, and the most salient of these were increases in excitatory FR response, which could come even from cells that did not respond to the lower concentration (Fig. 1*B*, cell a and cell b). To compare changes in response for higher concentration to those for faster sniffing, we took trials from each stimulus concentration based on inhalation duration: slow sniffing trials (for both high and low concentrations) were taken as the five trials with highest mean inhalation duration (MID), and fast sniffing trials were the five trials with the lowest MID. When comparing changes in FR evoked by concentration increase to those taking place as a result of increased sniff frequency, it was apparent that very similar changes took place (Fig. 1*B*). Altogether we recorded from 20 mitral/tufted cell-odor pairs in such a manner, with a range of FR responses to the low concentration odorant (Fig. 1*D*). When taking a broad measure of the change in firing rate across the first second of the stimulus (normalized by baseline SD), changes in FR were significantly correlated between those resulting from concentration increase and those resulting from faster sniffing (*R* ^2^ = 0.70, *p* = 5 × 10^−6^, *n* = 20; Fig. 1*C*). Furthermore, comparing heat maps of the changes in FR due to increased concentration and due to increased sniff frequency revealed a very similar set of changes that were significantly correlated compared to shuffle controls (*R* = 0.71, *p* = 5 × 10^−27^; *n* = 160 time bins; Fig. 1*E*,*F*; see Methods). This level of correlation was very similar to that for the FR changes due to concentration increase when compared between two random halves of high-concentration trials (*R* = 0.73, *p* = 6 × 10^−28^). Overall, this indicates that the pattern of FR changes across time bins was highly similar for concentration increase and for fast sniffing.

While in the output of MTCs the effects of sniffing and concentration increase were very similar, differences were seen in the subthreshold response changes, suggesting that changes in input in the two cases were (perhaps unsurprisingly) not identical: increases in inhibition were generally larger for the concentration increase than for faster sniffing (Fig. 1-3). We suggest this could be the result of inhibitory networks that act to normalize olfactory bulb output (within limits) in the face of increased global input (Kato et al., 2013; Miyamichi et al., 2013; Roland et al., 2016).

Thus, while increased concentration causes greater increases in subthreshold inhibition than increased sniff frequency, the latter results in changes in olfactory bulb output that apparently mimic those resulting from increases in concentration.

### Faster inhalation mimics effect of concentration increase on latency response in the timescale of a single sniff

It has been reported that increased concentration causes changes in response on finer temporal timescales, in particular the temporal advance of excitatory bursts (Cang and Isaacson, 2003; Fukunaga et al., 2012; Schaefer and Margrie, 2012; Sirotin et al., 2015). MCs undergo robust reductions in latency of excitation for concentration increase, while TCs—which already respond earlier—do not (Fukunaga et al., 2012). We wanted to know whether faster sniffing could cause the same temporal effects as concentration increase on a cell-by-cell basis.

To determine this, we first analyzed 13 cell-odor pairs with early excitatory responses (within 250 ms of odor onset) recorded in passive awake mice where only a single concentration stimulus (1% saturated vapor pressure) was presented to the animal across trials. Comparing the FR response over the first 250 ms for fast sniff trials (>70th percentile peak inhalation slopes) and slow sniff trials (<30th percentile), it was apparent that faster inhalation could cause a latency advance of the excitatory burst (Figs. 2*A* and 2-1*F*). Consistent with previous results (Carey and Wachowiak, 2011; Shusterman et al., 2011), faster inhalation caused a significant latency reduction in mean response onset across the dataset (latency change, fast-slow = –16 ± 14 ms, *p* = 0.002,^a^ *n* = 13, paired *t* test between onsets for slow and fast inhalations; Fig. 2-1*A*,*B*). Onset latencies displayed a significant relationship with the peak firing rate during the response (Fig. 2-1*D*), suggesting that the most strongly activated cells respond earlier. The extent of the latency reduction correlated with the onset time during slow inhalation: if the response was of longer latency during slow sniffing, the latency reduction was greater (Fig. 2-1*C*), indicating that cell-odor pairs showing a stable latency are likely already activated at the earliest possible timescale. We next used sniff cycle phase preference (calculated from *V_m_* during baseline breathing in air) to determine putative MC and TC (pMC and pTC) phenotype using subthreshold activity as previously described (Fukunaga et al., 2012; Jordan et al., 2018). Examples could be found where both pMCs and pTCs underwent reductions in latency of excitation when the sniff was fast (Fig. 2*A*); however, in general, reductions for pMCs were greater than reductions for pTCs (pMCs: latency change = –30 ± 7 ms, *p* = 7 × 10^−4^, paired *t* test,^b^ *n* = 5 cell-odor pairs; pTCs: latency change = –8 ± 10 ms, *p* = 0.08,^c^ paired *t* test, *n* = 8 cell-odor pairs; pMCs versus pTCs: *p* = 0.001,^d^ unpaired *t* test; Figs. 2*B* and 2-1*E*), and this was potentially because pTCs already tended to respond with shorter latency during slow sniffs than pMCs (Fig. 2*B*). Thus, the effect of fast sniffing, including cell-type specificity, is similar to that previously reported for increasing concentration (Fukunaga et al., 2012).

We next asked whether the effect of sniffing on latency directly mimics the effect of concentration change within a single cell. When comparing high- and low-concentration stimuli over the first 250 ms in MTC recordings from passive mice (dataset as in Fig. 1), the only salient changes in response to increased concentration were latency advances of excitatory burst stimuli (Fig. 2*C*,*D*). When correlating the pattern of changes in spike count as before (Fig. 1*F*) between those occurring for sniff change and those occurring for concentration change, there was a significant positive correlation between the two (*R* = 0.71, *p* = 4 × 10^−72^, *n* = 525 time bins; Fig. 2*E*). This level of correlation was only marginally smaller than that when correlating spike count changes due to concentration increase between two random halves of high-concentration trials (*R* = 0.78, *p* = 4 × 10^−111^). Latency reductions for concentration increase were similar in magnitude to those seen due to sniff change (Fig. 2*F*, mean onset advance = –18 ± 10 ms, *p* = 0.04,^e^ *n* = 4; paired *t* test between onsets for low and high concentration), and similar to those previously reported (Sirotin et al., 2015). To determine the effects of sniffing on ability to distinguish the two concentrations from our dataset, we calculated the Euclidean distance between FR responses to the two different stimuli. This revealed that latency changes contributed to the entirety of the difference between the two different concentrations on this timescale, with the Euclidean distance between the two dropping to baseline if the excitatory bursts were manually shifted forward for the low concentration (Fig. 2*G*, slow sniff vs slow sniff adv.; see Methods). Faster inhalations during low-concentration trials mimicked the latency shifts caused by concentration increase, also causing the Euclidean distance between high and low concentration stimuli to drop to baseline (Fig. 2*G*, slow sniff vs fast sniff).

Thus, even on short timescales, a more rapid inhalation mimics concentration increases at the level of the single-cell output from the OB, making it very difficult to distinguish the effect of increased concentration at this timescale.

### Variance in sniffing has no overt impact on performance in a fine concentration discrimination task

Rodents have previously demonstrated the ability to discriminate odor concentrations (Slotnick and Ptak, 1977; Abraham et al., 2004; Parthasarathy and Bhalla, 2013; Wojcik and Sirotin, 2014); however, it is not known how sniff variance affects this ability. Given the physiology (Figs. 1 and 2; accompanying paper, Shusterman et al., 2018), we next sought to determine the capabilities of mice when distinguishing odor concentrations in a simple head-fixed go/no-go paradigm (Fig. 3*A–C*), despite variance in sniffing.

First, mice were trained to distinguish high-concentration (3%) versus low-concentration (1%) stimuli. Three mice were trained with the low-concentration stimulus as the CS+ (Low go), and five mice were trained with high concentration as the CS+ (High go). To ensure mice could not use flow changes to perform the task, our olfactometer design kept flow from odor outlet constant (Fig. 3-1*A*). After pretraining (Fig. 3-1*B*), all mice learned this task within a single training session (Fig. 3*D*) and could make rapid decisions within the timescale of a single sniff cycle (160–200 ms; Fig. 3*E*). To test whether mice were using trigeminal rather than olfactory input, after the task was learned, the odor would subsequently be switched to vanillin (a chemical that is thought not to stimulate trigeminal afferents; Frasnelli et al., 2011), presented at the same two concentrations. Mice learned to perform this discrimination within a significantly shorter time frame than the original odor mixture, and with the same short reaction times (Fig. 3*D*,*E*). This suggests the mice may have learned the task rule for odor concentration and applied it rapidly to the new, non–trigeminal-activating odorant. Learning in the task was likely the result of acquiring the response to the stimulus rather than learning how to perceive the difference in concentrations, since on the very first presentation of the CS– concentration after pretraining on the CS+ concentration, mice typically displayed a rapid sniffing response (Fig. 3*F*) classically associated with stimulus novelty (Verhagen et al., 2007; Wesson et al., 2008b; Roland et al., 2016). Thus, in this task mice can very rapidly make decisions based on relatively modest concentration differences within the timescale of a single sniff, comparing very well to their abilities in odor identity tasks (Uchida and Mainen, 2003; Wesson et al., 2009; Resulaj and Rinberg, 2015).

To determine if sniff variation impacted the concentration decisions of mice, seven trained mice were advanced on to a five-concentration task. Here, three new intermediate concentrations between the two previously learned concentrations were also presented (Fig. 4*A*). The concentration most similar to the learned CS+ was rewarded as a CS+, while the other two concentrations, including one exactly halfway between the previously learned concentrations, were treated as CS– (Fig. 4*A*). Two to three sessions of 200 trials were performed on this task, over which mice generally performed at a relatively high level of accuracy (Fig. 4*B*,*C*, mean percentage correct across session = 75 ± 6%, *n* = 7 mice).

Was there any evidence that mice were learning a stable sniffing strategy to perform the task? This seems unlikely, as variance in inhalation duration of the first sniff did not decrease across the session (if anything, a mild increase in variance was observed: *R* ^2^ = 0.5, *p* = 0.0001, regression between block number and mean variance, *n* = 20 blocks; Fig. 4*D*,*E*), and variance was significantly larger across blocks compared to passively exposed mice (concentration go/no-go: standard deviation of 1^st^ inhalation duration = 19 ± 4 ms across 7 mice and 20 blocks; passive: SD = 13 ± 1 ms across 23 mice and 6 blocks; *p* = 0.004,^f^ *F* = 12.5, df = 1; two-way ANOVA; Fig. 4*D*,*E*). Thus, while mice are capable of more stable sniffing as seen during passive exposure, they do not use this as a strategy in the concentration task.

Mice displayed a graded percentage of go trials across concentrations, indicating that the discrimination task was not trivial (Fig. 4*B*). Thus, if sniff changes cause shifts in perceived concentration, this should be overtly seen in the performance curves, and importantly, these shifts should have opposite polarity depending on which contingency the mouse was trained on (i.e., fast sniffing for Low go trained mice should decrease go responses, while it should increase go responses in High go trained mice). To test this, we first separated trials according to whether the first sniff was fast (<30^th^ percentile inhalation duration) or slow (>70^th^ percentile; Fig. 4*F*). This resulted in a comparison of trials between which the difference in the inhalation duration exceeded that used in the whole-cell recordings when comparing fast and slow sniff trials (Fig. 3-1*C*). Calculating performance curves separately for fast and slow sniff trials for each mouse revealed that there was a significant tendency for higher go rates in fast sniff trials for both Low go and High go trained animals across concentrations (Low go: *p* = 0.04,^g^ *F* = 4.8; High go: *p* = 0.001,^h^ *F* = 14.1; three-way ANOVA). However, this tendency did not differ across concentrations or training contingency (two-way ANOVA performed on difference in go rate between fast and low sniff trials across mice: contingency versus go rate difference: *p* = 0.41^i^; *n* = 7 mice × 5 concentrations; Fig. 4*G*). This makes it more likely that a fast sniff indicates higher motivation to do the task (consistent with previous findings; Wesson et al., 2009; Jordan et al., 2018), resulting in higher go rates across the board.

Thus, we wanted to more directly probe the effect of sniff variance on performance. On a small selection of trials for five of the mice, the puff stimulus (as used during the physiologic recordings) was used to evoke fast sniffs, including the first inhalation (Fig. 4*J*). The mean changes in first inhalation duration evoked by this puff were again highly comparable to that used for analysis of fast and slow sniffs in the physiologic data (Fig. 3-1*C*). The puff was associated with an increased error rate likely owing to distraction, but this did not reach significance (percentage correct: control trials = 83 ± 8%, probe trials = 77 ± 9%, *p* = 0.16,^j^ paired *t* test, *n* = 5 mice). There was a small and insignificant tendency for increased go rates during the puff stimulus relative to control trials for mice trained on either contingency (Low go: *p* = 0.17,^k^ *F* = 2.0, df = 1; High go: *p* = 0.14,^l^ *F* = 2.3, df = 1; three-way ANOVA performed on go rate; Fig. 4*K*), and this tendency was not significantly different between mice trained on the two contingencies (*p* = 0.84,^m^ *F* = 0.04, df = 1; two-way ANOVA performed on difference in go rate between fast and low sniff trials across mice).

Could mice be compensating for ambiguity by taking more inhalations to make the correct response? If so, this would be reflected in longer reaction times for fast compared to slow first sniff trials. On the contrary, comparing trials with fast and slow inhalations as above (Fig. 4*F*), reaction times (calculated between the highest and lowest concentration) were slightly though significantly shorter for fast sniff trials (Δreaction time, fast-slow = –35 ± 38 ms, *p* = 0.048,^n^ paired *t* test, *n* = 7, Fig. 4*H*), again consistent with the idea that faster sniffing indicates a higher motivation level (Wesson et al., 2009; Jordan et al., 2018). Reaction times were unaffected by the puff stimulus compared to control trials (Δreaction time, probe-control = –5 ± 45 ms, *p* = 0.80,^o^ paired *t* test, *n* = 5, Fig. 4*L*). This was also the case for finer concentration discrimination (Fig. 3-1*D*,*E*).

Reductions of inhalation duration of 10–20 ms rendered 1% and 2.5% concentrations hard to distinguish within our sample of MTC cells (Fig. 2G). Here we are comparing similar and even larger reductions in inhalation duration, yet behaviorally the ability to discriminate concentration on an even finer scale shows no overt differences, congruent with findings in rats for a different task in an accompanying paper (Shusterman et al., 2018). Thus, mice can easily discriminate fine concentration differences even in the face of large changes in sniffing.

### Mitral and tufted cells respond to inhalation changes in absence of applied odor

We have so far shown that it is difficult to distinguish the effect of a change in inhalation or a change in concentration via their effects on MTC responses (Figs. 1 and 2), yet mice are perfectly capable of fine concentration discrimination in the face of fluctuating inhalations (Fig. 4). One explanation for this apparent conundrum could be that the olfactory system obtains information about the kind of inhalation that just occurred to infer whether concentration or sniffing evoked the response change. Congruent with the latter idea, OSNs have been demonstrated to respond to pressure changes (Grosmaitre et al., 2007), giving rise to sniff coupling in the olfactory bulb (Adrian, 1950; Macrides and Chorover, 1972; Cang and Isaacson, 2003; Margrie and Schaefer, 2003; Fukunaga et al., 2012), which disappears with naris occlusion (Margrie and Schaefer, 2003), and bouts of rapid sniffing are known to cause activity changes in MTCs in absence of applied odor (Jordan et al., 2018; Kato et al., 2013). We wanted to determine if the olfactory bulb reports graded changes in inhalation parameters on the timescale of a single sniff.

**Figure 5.**
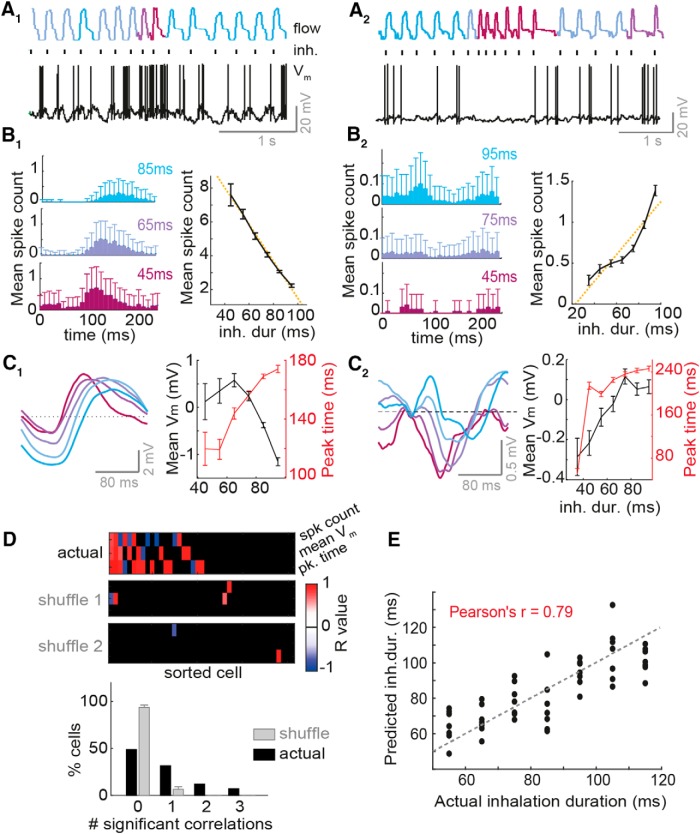
Inhalation duration transforms mean baseline MTC activity in a large proportion of cells. ***A_1_–C_1_*** refer to one example cell, while ***A_2_–C_2_*** refer to a different example cell. ***A_1–2_***, Example nasal flow traces and *V_m_* traces in absence of odor. Sniffs are color coded according to inhalation duration (blue = slow, and red = fast). Black ticks indicate inhalation onset. ***B_1–2_***, Left: average spike count histograms triggered by inhalations of different durations (denoted on each histogram). Right: mean spike count per sniff as a function of inhalation duration. Error bars = standard error (SE). ***C_1–2_***, Left: inhalation-triggered mean *V_m_* waveforms for sniff cycles of different inhalation duration. Right: mean *V_m_* and timing of *V_m_* peak for membrane potential waveforms averaged across all sniffs as a function of inhalation duration. Error bars = SE. ***D***, Top: heatmap of *R* values for correlations between inhalation duration and 3 different activity parameters (spike count, mean membrane potential, and timing of peak membrane potential, rows 1–3, respectively), for 45 MTCs. Cells are sorted left to right from largest number of significant correlations to lowest number. Black squares show where the correlation was insignificant (*p* > 0.01, linear regression). Two lowest heatmaps show the same data but for two example shuffle controls, where inhalation durations were shuffled with respect to the physiology, and the data reanalyzed. This gives an indication of false-positive rates in this analysis. Bottom: histogram to show proportion of cells with 0–3 significant correlations between the different activity parameters and inhalation duration. Gray shows proportion for shuffle controls. ***E***, Scatter plot between inhalation duration predicted by a simple linear model using peak spike rates of 25 cells (see Methods) and the actual (true) inhalation duration for all 7 sniff cycles tested in each category. See Fig. 5-1 for the impact of cell type on responses to inhalation change, Fig. 5-2 for further analysis regarding detecting inhalation change, and Fig. 5-3 for a hypothetical relative timing code using this information to infer environmental concentration change.

We took baseline activity in absence of odor as a proxy for the large portion of mitral and tufted cells that will not be responsive to an odor, whose activity could instead be used to directly determine the kind of sniff that took place. To do this, we analyzed the cellular activity of 45 MTCs recorded in passive mice across over 1000–2000 sniffs occurring in absence of the odor. Sniffs were categorized according to inhalation duration, and for each category peristimulus time histograms and average membrane potential waveforms were calculated over 250 ms triggered by inhalation onset (Fig. 5*A–C*). We found that individual MTCs would show linear transformations in their activity according to the duration of the inhalation just occurring. For example, some cells showed increased spike count (Fig. 5*A*_1_,*B*_1_) and depolarizing membrane potential (Fig. 5*C*_1_) as inhalations became faster, while others showed decreasing spike count (Fig. 5*A*_2_,*B*_2_) and more hyperpolarizing membrane potential (Fig. 5*C*_2_). 24% of cells showed significant relationships between spike count and inhalation duration (*p* < 0.01, linear regression; Fig. 5*D*) compared to only 3% in shuffle controls (odds ratio = 7.8, *p* = 1 × 10^−5^,^p^ Fisher’s exact test). Similarly, 22% showed significant correlations with mean membrane potential compared to 2% of shuffle controls (odds ratio = 9, *p* = 3 × 10^−5^,^q^ Fisher’s exact test; Fig. 5*D*). Timing of activity was also often linearly correlated with inhalation duration, generally with the peak of the membrane potential shifting to earlier times as inhalation duration reduced (significant *R* values in 32% of cells versus 2% in shuffle controls, odds ratio = 14, *p* = 1 × 10^−8^,^r^ Fisher’s exact test; Fig. 5*D*). Altogether 51% of cells showed a significant relationship between inhalation duration and at least one or more of these activity parameters (Fig. 5*D*). Interestingly, the directionality of the relationships (i.e., whether a cell hyperpolarizes or depolarizes with a faster sniff), could be attributed to putative mitral or tufted cell type, as defined by sniff phase preferences (Fig. 5-1).

10.1523/ENEURO.0148-18.2018.f5-1 Extended Figure 5-1 Cell type specificity of effect of inhalation as defined by sniff-phase preference. In absence of applied odor, putative mitral cells (pMCs) respond to faster sniffs with increases in inhibition, and putative tufted cells (pTCs) with increases in excitation. (A1) Reconstructed morphology of a tufted cell recorded in awake mouse. 'Bb' refers to brain border, 'EPL' refers to external plexiform layer and 'MCL' refers to mitral cell layer (these morphologies have been previously published in Jordan et al. 2018 for different purposes). (B1) Example nasal flow and Vm trace during a rapid sniff bout (blue to purple represents longer to shorter inhalation duration on flow trace. Spikes have been cropped for display. (C1) Mean membrane potential waveform for different bands of inhalation duration: blue = long inhalation duration, purple = short. (A2)-(C2) as for A1-C1, but for a filled mitral cell recorded in an awake mouse. (D) R values for correlations between inhalation duration and mean Vm as a function of phase preference. Only strong correlations have been included (p<0.05 and R2>0.6). Grey line shows mean R value for all cells within a 2 radian moving window (centred), to give an idea of the phase modulation strength of the data. There was a significant organisation according to phase (p<0.01, bootstrapping, see methods). Boxplots to the right compare all values within the putative MC (red) and putative TC (blue) phase boundaries (mean Vm: pMC: median = 0.93, IQR = 0.84 to 0.95, n = 6; pTC: median = -0.83, IQR = -0.96 to -0.82, n = 10; p = 0.002, Ranksum). These phase boundaries are based upon those used in previous studies (Jordan et al., 2018; Fukunaga et al., 2012). (E) As for panel D, but for mean spike count per sniff. Again, there was a significant organisation according to phase preference (p<0.001; bootstrapping, see methods), and R values were significantly different between pMC and pTC boundaries (spike count: pMC: median = 0.84, IQR = -0.88 to 0.96, n = 22; pTC: median = -0.92, IQR = -0.94 to -0.89, n = 12; p = 0.008, Ranksum). Download Extended Figure 5-1, TIF file.

10.1523/ENEURO.0148-18.2018.f5-2 Extended Figure 5-2 Detection of inhalation duration change and cell 'weights' in linear model (A) Top: diagram to show construction of sniff sequences of different inhalation duration: either three of 95 ms inhalation duration (blue), or two of 95 ms with the final sniff of 55 ms duration (purple). Below shows two example PSTH sequences (from two different cells) averaged from random subsets of 25 sniffs that show the particular inhalation duration. Blue plot shows the PSTH sequence for 4 sniffs of 95 ms, and purple plot shows sequence in which the last inhalation is of 55 ms. Bottom trace shows mean Euclidean distance calculated between population vectors containing all cells constructed from the two sniff sequences as in panel A. Plot shows the average of 5 different subsets of data (made by averaging different sniff subsets for each cell), and shaded area shows standard deviation. Dashed red line indicates time of significant detection of change. (B) As for panel A, this time comparing PSTHs for a smaller inhalation duration change (95 ms, blue to 75 ms, purple). (C) Regression coefficients (weights) for individual MTCs in the linear model used to predict inhalation duration based on peak spike rates (related to Figure 5E; see methods). Download Extended Figure 5-2, TIF file.

10.1523/ENEURO.0148-18.2018.f5-3 Extended Figure 5-3 Diagram of a potential relative timing code for concentration. (A) Highly simplified diagram of the olfactory bulb, depicting only glomeruli and MTCs. Green glomeruli/MTCs show those receiving odor input (odor responsive), while grey glomeruli/MTCs show those with absence of such input (unresponsive population). While odor inputs are sparse, mechanical input (response to the pressure change associated with each sniff) is widespread. (B) Diagram to show how a relative time code may work. Each instance shows one sniff prior to and during odor stimulation. 'Nasal flow' shows these two sniffs, with grey showing slow sniffs (>95 ms inhalation duration) and red showing a fast sniff (e.g. 55 ms inhalation duration). 'Odor concentration' trace shows a step increase in concentration from zero just prior to the second sniff. Grey traces show low concentration and red shows high concentration (2 x low concentration). 'Unresponsive population' shows a hypothetical population average FR for all cells without odor inputs during the odor stimulus. Dotted traces in second and third column for the second sniff cycle show the trace for the first column, for sake of comparison. 'Odor responsive' shows the average population FR for cells receiving direct odor input. Dotted traces in second and third column for the second sniff cycle show the trace for the first column, for sake of comparison. Δt shows the difference in peak population activity for unresponsive and odor-responsive cells during the stimulus. Note that Δt remains stable unless the concentration changes, and not when the sniff alone changes. A change in Δt allows perception of a different concentration. Download Extended Figure 503, TIF file.

We next sought to determine if we could detect changes in inhalation from the spiking activity of cells in absence of odor. For all cells with enough sniff variation (>50 sniffs in each inhalation duration category: 95–105, 55–65, and 75–85 ms), we calculated sequences of spike histograms for sniffs of different inhalation durations using random subsets of sniffs within each category. We constructed either a sequence with PSTHs calculated from three consecutive sniffs of 95-ms inhalation duration or a sequence with PSTHs calculated from 2 consecutive sniffs of 95 ms, with the last PSTH instead constructed from 55-ms inhalation duration sniffs (Fig. 5-2*A*). Using these, it was possible to determine a change in inhalation duration (95–55-ms inhalation duration) within only 70 ± 12 ms by calculating Euclidean distances between constructed population vectors of the two different sequences (Fig. 5-2*A*). Smaller changes in inhalation duration (95–75 ms) could also be detected on similarly rapid timescales (Fig. 5-2*B*).

We next wanted to assess the overall predictive power of MTC firing activity for inhalation duration. Using 25 whole-cell MTC recordings, we generated a simple linear model to classify inhalation durations within 10-ms bins using the peak spike rates within each sniff cycle. The linear model was generated using constructed “population” activity of the 25 cells across 13 sniff cycles from each inhalation duration category and was tested subsequently on 7 sniff cycles from each category. Considering the limited number of cells and trials used, this classifier performed very well (Pearson’s *r* = 0.79; Fig. 5*E*). Comparing the model’s weights (regression coefficients) for different cells, we found that the large majority of cells were involved in the classification, but weights for pMCs tended to be stronger than for pTCs and showed significantly larger variance (*p* = 0.01, Bartlett test; Fig. 5-2*C*).

Thus MTC activity—in the absence of applied odor input—is informative of the inhalation that just occurred, such that the large population of non–odor-responsive cells could be utilized by the olfactory system to distinguish sniff changes versus concentration changes.

## Discussion

For stable perception, sensory systems must find ways of encoding of stimulus features independent of fluctuating sampling behaviors, such as eye movements or sniffing. Here we show that faster sniffs can evoke response changes in the olfactory bulb that appear indistinguishable from those caused by increasing concentration (Figs. 1 and 2), yet mice are highly capable of perceiving concentration on fast timescales, regardless of sniffing parameters (Fig. 4). We reason that a way the olfactory system could distinguish these two occurrences directly is via information about the kind of sniff that just occurred. While this could conceivably happen downstream via efference copy of sniff motor commands, we find that MTC activity already allows inference about the kind of sniff that just occurred on a rapid timescale (Fig. 5). Thus, the olfactory bulb itself does not appear to be the site where the sniff-invariant percept of intensity is generated, but does appear to already contain information that could be used to generate the percept elsewhere.

Given the timescale of decision-making for concentration (Figs. 3 and 4), it seems likely that the information used by the mouse is the fast-timescale temporal shifts in excitation that have been previously described (Cang and Isaacson, 2003; Fukunaga et al., 2012; Sirotin et al., 2015). Congruently, this temporal information contributes to the entirety of the difference in response to the two concentration stimuli in our dataset (Fig. 2*G*). It has been suggested that high baseline firing rates of MTCs could obscure such a latency code for concentration being used (Mainland et al., 2014); however, this was based on a overestimation of baseline FRs from unit recordings. The whole-cell recordings we employ here are thought to be unbiased in terms of baseline FRs (Margrie et al., 2002; Shoham et al., 2006; Kollo et al., 2014), and discriminability of MTC responses based on latency shifts is overt (Fig. 2G). Congruently it is known that mice can perceive the latency difference in optogenetic glomerular activation on the order of tens of milliseconds (Smear et al., 2013; Rebello et al., 2014).

Sniff changes have been hypothesized to alter odor concentration profiles within the nasal cavity (Teghtsoonian et al., 1978; Shusterman et al., 2018). Here we show for the first time directly that sniff changes can indeed mimic the effect of concentration change at the level of both firing rates (Fig. 1) and temporal shifts in spike activity (Fig. 2). This is not to say that OSN input is perfectly matched when we compare faster sniff rates and higher concentration. In fact, since subthreshold inhibition is greater for the higher concentration (Fig. 1-3), it would appear that the input strength is higher for the case of increased concentration as compared to faster sniffing. Despite this, overt changes in the spiking output are very similar for increased sniff frequency compared to increased concentration. Potentially, inhibitory circuits are normalizing the spiking output across large changes in input (within a dynamic range), such that while we see differences in subthreshold inhibition, the excitatory spike outputs look very similar. Such a role has been suggested for the various external plexiform layer and juxtaglomerular cells (Tavakoli et al., 2018), including periglomerular (Aungst et al., 2003; Roland et al., 2016), dopaminergic (Banerjee et al., 2015), and parvalbumin-positive (Kato et al., 2013; Miyamichi et al., 2013) neurons.

Here we chose relatively high concentrations (1%–3% saturated vapor pressure) to ensure a good rate of response in whole-cell recordings, and relatively modest concentration differences (up to 0.5 logfold change), since we expected sniff-related differences in representation to have the most pronounced effect on performance in these fine discriminations. The question then arises, at what concentration range will sniff variation affect concentration estimation (at the level of OB activity)? This is difficult to answer without direct measurement of naris odor concentration, but we can make some tentative hypotheses. Fluid dynamic models predict that a sniff with a higher flow rate will cause the temporal profile of concentration in the naris to become steeper—i.e., more odor molecules are drawn in per unit time (Shusterman et al., 2018). Thus we would expect the concentration change that a sniff change can mimic to be proportional to both the environmental concentration and the range over which nasal flow can change (previous measurements show that this is at least twofold; e.g., Youngentob et al., 1987).

It has been known for some time that the olfactory bulb is highly modulated by the sniff cycle (Adrian, 1950; Macrides and Chorover, 1972; Cang and Isaacson, 2003; Margrie and Schaefer, 2003; Wachowiak, 2011; Fukunaga et al., 2012). Since sniff modulation is more overt in anesthetized mice and is seemingly reduced at higher sniff frequencies (Kay and Laurent, 1999; Bathellier et al., 2008; Carey and Wachowiak, 2011), the importance of sniff modulation in the awake animal may come into question. Here we find that sniff patterning of activity in awake mice gives rise to linear transformations of baseline activity as inhalation parameters are changed, a feature that is widespread throughout MTCs (Fig. 5). We thus reason that a key function of sniff modulation could be to inform the olfactory system about what kind of inhalation took place, such that a change in concentration and a change in sniffing are readily distinguishable without explicit information from breathing control centers. Congruently, we find that inhalation parameters can indeed be readily and rapidly inferred from the spiking activity of MTCs (Figs. 5*E* and 5-2).

Sensory encoding of sniff parameters has been hypothesized previously when psychophysics showed that humans could categorize concentrations well despite large changes in inhalation flow rate (Teghtsoonian et al., 1978). Previous work has shown that sniff modulation of the olfactory bulb is generated predominantly peripherally rather than centrally, since blocking the naris abolishes sniff modulation in the olfactory bulb (Sobel and Tank, 1993; Margrie and Schaefer, 2003; Schaefer et al., 2006; Iwata et al., 2017). One possibility is that the olfactory system uses reafference (the sensory effect of the sniff) to infer the kind of flow rate evoked by the sniff, and thus determine real changes in concentration from those caused by flow changes. This could be an explanation for the observation that olfactory receptors respond to pressure changes as well as olfactory stimuli (Connelly et al., 2015; Grosmaitre et al., 2007), and indeed may comprise a feature rather than a bug in the olfactory system. Consistently, concentration perception in humans can be affected when the nostril flow rate was changed via experimenter-induced changes in airway resistance instead of volitional changes in sniff pressure (Teghtsoonian and Teghtsoonian, 1984)—i.e., only when flow rate is altered but pressure stays constant. Moreover, imaging of the olfactory cortex in humans identified a region that primarily responds to the sensory effect of sniffing in absence of odor (Sobel et al., 1998). However, it is possible that the system employs predictive coding (Wolpert et al., 1995), in which an internal model of the respiratory motor system predicts the effect on nasal odor concentration based on the sniff command, and accounts for this somewhere in the olfactory pathway. Since airway resistance is subject to continual changes and even differs between the two nostrils (Principato and Ozenberger, 1970; Sobel et al., 1999), the internal model would require constant updating individually for each nostril, and mechanical sensory reafference from mitral and tufted cells could be used to do this on a sniff-by-sniff basis. However, since there is currently no known projection from the sniffing motor system to the olfactory system, and given that mitral and tufted cells can detect a sniff change on rapid, behaviorally relevant timescales (Fig. 5-2), a purely feedforward solution could be an efficient way to encode sniff effort.

An accompanying study intuitively suggests that the advance of odor-driven excitation as sniff frequency increases is the result of fluid dynamics in the nasal cavity (Shusterman et al., 2018). While we do not investigate the coding scheme used for invariant concentration coding, the accompanying study examines various models in detail. A large fraction of our cells show an advance of their baseline activity peak as the inhalation becomes faster (Fig. 5*D*). We could thus hypothesize that non–odor-responsive MTCs within a region of the bulb can provide information about the timing of inspired air reaching the epithelium. If the inhalation becomes faster, both responsive and the much larger population of unresponsive cells show a latency reduction in their peak activity, while if concentration has increased, only the sparse odor-responsive population will show this latency shift. Thus, a relative timing code could be used as a sniff-invariant representation of concentration (Fig. 5-3). Previous imaging work congruently suggests that subtracting the population response of MTCs throughout the entire bulb can act to provide more consistent trial-to-trial odor responses and remove variation associated with sniffing (Blauvelt et al., 2013). Exactly where and how the two kinds of information could be integrated to form a sniff-invariant representation of concentration should be the objective of future investigations, though recent evidence from the piriform cortex of awake mice already suggests that cortical interneurons sharpen the latency shifts evoked by concentration change and encode concentration via the synchronicity of ensemble firing (Bolding and Franks, 2017). It is possible that in a mouse performing a concentration guided task, even the olfactory bulb circuit could be altered by top-down circuits in such a way as to generate a sniff-invariant representation of concentration using information about the sniff dynamics.

Our results show that for response latency or FR on the single-cell level, fast sniffing at low concentration looks very similar to slow sniffing at high concentration. We hypothesize that the mechanism for the reduced latency of response for both increased nasal flow and increased concentration is similar—in both cases, the concentration profile in the naris is steeper, and OSNs depolarize to threshold more rapidly. At the lower end of the concentration scale, this would even occur for the most highly sensitive “first responding” MTCs that have been hypothesized to account for sniff- and concentration-invariant odor identity codes (Wilson et al., 2017). We would thus predict that, for single-cell latency or FR responses, even a large population of cells similar in properties to those recorded here would not help distinguish the two scenarios. It is possible, however, that there is a small and specialized MTC subtype that might encode odor concentration in a simple, sniff-invariant manner. We deem this less likely, since the accompanying study records from a much larger portion of MTCs and finds that sniff variance still renders concentrations very difficult to discriminate (Shusterman et al., 2018). Alternatively, population level codes could be employed for concentration encoding (e.g., via spike synchrony or higher-order features), which are robust in the face of sniff change but elude identification with single-cell recordings. It is also possible that sniff-invariant features appear in the OB after training on the concentration task; however, it must be noted that, in our hands, our mice could detect even the relatively small difference in concentration within the first presentation of the novel stimulus (Fig. 3*F*), suggesting that fine odor discrimination occurs readily in a sniff-independent manner, not requiring any extensive training.

In conclusion, concentration changes in the naris can be either self-generated through changes in sniffing or the consequence of a true change in environmental concentration, yet mice can perform sniff-invariant concentration discrimination. The olfactory bulb contains information about the odor concentration alongside the inhalation dynamics, which together may allow inference about whether a sniff change or a concentration change occurred, overall enabling sniff-invariant concentration perception.

## Synthesis

Reviewing Editor: Anna Menini, SISSA (International School of Advances Studies) Decisions are customarily a result of the Reviewing Editor and the peer reviewers coming together and discussing their recommendations until a consensus is reached. When revisions are invited, a fact-based synthesis statement explaining their decision and outlining what is needed to prepare a revision will be listed below. The following reviewer(s) agreed to reveal their identity: Sandeep Datta.

This is the first real analysis of the impact of sniff rate variability on MT cell encoding in the awake animal that directly compares its effects to those observed with changes in concentration - addresses a very general problem about perceptual stability in sensory systems and proposes an interesting model.

Both reviewers agree that the experiments are impressive and appear well performed. The reviewers have specific suggestions for improving the manuscript related to data analysis, interpretation and discussion. Please respond to the comments of each reviewer as detailed below and make appropriate revisions to your manuscript.

Reviewer #1

In this manuscript, the authors examine the interaction between ‘rapid sniffing’ in awake mice and concentration-invariant coding of odor identity, using a combination of whole-cell recordings and behavioral assays. They find that rapid sniffing has a similar impact as increasing odor concentration on mitral and tufted cell responses, although in separate behavioral assays, sniffing behavior does not correlate with discrimination of different-concentration odors. They then provide evidence that this apparent confound can be explained by decoding sniff parameters from respiration-patterned activity across a population of mitral and tufted cells. While I have some reservations about the strength of their proposed model, it is an important idea and the study, on the whole, involves an extremely impressive set of experiments and contains a wealth of interesting data which are important for driving further exploration of these ideas. I congratulate the authors on this impressive work. There are a few important points regarding analysis and interpretation points that should be addressed, however, and discussion of these results in the context of prior studies could be improved, as detailed below.

Major points

1. One technical concern is that, as far I understand it, all analyses comparing concentration and sniff speed effects on firing rate and latency come from averages across multiple trials. The number of trials used for this averaging are not clear. For example, for the data shown and discussed in Figure 1C, D and E, from how many trials are firing rates averaged, for each cell? Likewise, for the latency data shown in Figure 2 and related text, how many responses were averaged to generate FR profiles in order to calculate response latency? This is important because of the possibility that some of the apparent effects of fast sniffing could result solely from averaging trials - for example, an increased reliability (or decreased baseline variance) from trial to trial during high frequency sniffing would be expected to generate stronger and faster average responses even when such an effect is absent on a single-trial basis. If there were a reasonable way to assess the impacts of fast and slow sniffing, and high and low concentrations, on a trial-by-trial basis (which is the situation the animal is faced with in reality) this would strengthen the overall conclusions.

2. The odor concentrations used are, first, both quite high (in this reviewer's opinion) and, second, not all that different from one another: a 2.5-fold change in concentration (which is only 0.4 log unit change in intensity) does not present a particularly challenging problem in terms of concentration-invariant odor perception. This is relevant for alternate interpretations of the authors' results, as detailed in comment 3 below. Do the authors suspect that sniff speed mimics changes in odor concentration only over this modest range? Over what range do odor concentrations vary in a naturalistic plume? Addressing these questions in their Discussion would be helpful for thinking about the relevance of their model for behavior under naturalistic conditions.

3. The behavioral data presented in the middle section are very nice and convincingly suggest that sniff speed (specifically, inhalation duration) is not a confound in discriminating different-concentrations. These results are used as the justification to search for mechanisms by which the ‘paradox’ of sniff-dependent changes in mitral/tufted cell responses is resolved - namely, the ‘reading’ of sniff parameters from a population of sniff-driven mitral/tufted cells, which is explored in the final section. There is nothing wrong with this idea, but to this reviewer there is a simpler explanation, which is that the mitral/tufted cell population code discriminating the different learned concentrations is sufficiently robust that it is not perturbed by the rather modest changes in firing rate or small changes in latency that accompany differences in sniffing. This could especially be true after the extensive training involved here, and if one considers the large numbers of neurons (far more than are recorded by the experimenters) that are available to contribute to robust odor identity coding. In short, the ‘paradox’ the authors nicely explain away may not be much of a paradox at all.

I do not mean to invalidate the final section of the study, which is quite nice in its own right, but this alternative explanation should at least be acknowledged.

4. There are a number of points in this paper which were addressed also in a recent publication from Diaz-Quesada et al. (J Neurosci 2018), yet this paper is not mentioned. It would be useful to note which results are similar to those reported in that study, and which are different. For example, the Diaz-Quesada study reported mixed effects of concentration on mitral or tufted cell response latency or amplitude, and a lack of correlation between effects of sniff speed and mitral versus tufted cell subtypes. Could these differences be related to recordings performed in awake versus anesthetized mice? Are there other explanations?

Minor comments.

5. The Results relating to differential effects of rapid sniffing on putative mitral or tufted cells are confusing and somewhat circular. First, the epoch used to determine phase preference for cell type classification (line 395) is not stated; was this during odor presentation or during baseline respiration of air? More importantly, the fact that pTCs show less reduction in latency with fast sniffing than do pMCs is not surprising, since pTC responses are already at a minimal latency by definition (this is why they are labelled pTCs). This should be acknowledged.

6. Details of how ‘nasal flow’ was recorded are missing. What sensor was used? Was this recorded external to or internal to the naris? Was this an actual flow sensor or simply a pressure sensor in which flow is inferred? These details are relevant to understanding how sniff parameters are derived for their analyses.

7. In Extended Data 1-2 (correlation of different sniff parameters), panel C appears to show that, at least in this animal, at sniff durations above approx 250 msec (4 Hz sniff frequency), there is no relationship between sniff duration and inhalation duration. For durations below 250 msec, a significant correlation is expected due to constraints of duty cycle for inspiration and exhalation. Thus, in my opinion the ‘significant’ correlation reported here is not informative.

8. The following text, as written, does not make sense: “Sniff cycles from 42 recorded neurons (25 MC, 17 TC) were divided into fast (37-80 ms), medium (80-96 ms) and slow (96-183 ms) cohorts. All cycles with sniff duration below the 0.5th percentile (108 ms) and above the 99.5th percentile (597 ms) were discarded. ”

I believe the authors are referring to inhalation duration in the first sentence when defining fast, medium and slow cohorts, not sniff cycle duration as is implied. Please clarify.

Reviewer #2

The paper entitled “Sniffing fast....” explores the inter-relationships between concentration and sniff rate, and asks about their relative effects on both MT cell firing and concentration discrimination in awake mice. The paper finds that changes in sniff rate mimic the effects of concentration changes on firing rates and dynamics (although not necessarily at the subthreshold level), but that the brain could potentially take advantage of the broad changes in firing rates apparent in odor-non-responsive neurons to know whether a given sniff was fast or slow, and thereby marginalize out the temporal effects of sniffing to enable concentration-invariant odor identity encoding. This paper is very carefully done, the similarities between changes in concentration and sniffing are eerie (and therefore provocative) and the analyses are convincing - it will be an important contribution to the field, and it offers an interesting feed-forward model for the conceptual problem of stimulus invariance. I have only a few minor comments regarding the manuscript:

1. Many of the conclusions rest upon figure 2G, in particular those about ‘discriminability.’ However no direct analyses of discriminability (from a classification or an information-theoretic perspective) are actually performed. Given the data in the figure this should trivially work if there is enough data, and I'm assuming this type of analysis was not included because of the relatively small number of cell-odor pairs included in the analysis, but if possible some additional formalization of this claim would be helpful. This is not critical to the paper - just a suggestion.

2. Similarly, with the decoding network in figure 5, it is not obvious which neurons you actually need to do the decoding, nor is it really clear whether the best you can do is binary inhalation speed classification, an important point since the distribution of sniff speeds isn't bimodal. Some simple playing with off the shelf SVMs here could strengthen this argument by allowing better fractionation of sniff rates (and maybe identify specific cells that are particularly good at carrying this kind of information). Given that you are looking at odor-non-responsive cells here, you might have enough data for this sort of analysis but, again, if you don't have enough data this is just meant as a suggestion.

3. The one conceptual concern here is that there are small populations of glomerulus-attached MT cells that, because they are connected to high-affinity receptors for a particular odor, operate in a quantitatively different regime than the remainder of the MT cells in the bulb. My superficial read of the recent Nature Comms Rinberg et al paper, which uses the high-affinity ligand for M72, suggests that these neurons might fire sooner than the distribution reported here for slow sniffs; it is formally possible, then, that these neurons could be report concentration changes on the low end in a manner that is invariant to sniff changes. The paper name-checks this idea in the discussion by saying that you might need to look at more neurons to be really sure that you aren't missing anything, but a couple more sentences about this specific possibility would make this caveat more clear (and wouldnot really take anything away from the paper - the fact that you can decode sniff rate from non-responsive neurons is incredibly important whether or not there is the ability to discriminate concentrations at the low end built into the system, and of course this high-affinity mechanism wouldn't apply to high concentrations, where perceptual invariance still applies and is a problem).

4. The legend to 5B threw me - because 5B2 includes the phrase “per sniff” but 5B1 does not I though these were different (and strange) analyses of the same data, rather than of two different cells.

## Author Response

Synthesis Statement for Author (Required):

This is the first real analysis of the impact of sniff rate variability on MT cell encoding in the awake animal that directly compares its effects to those observed with changes in concentration - addresses a very general problem about perceptual stability in sensory systems and proposes an interesting model.

Both reviewers agree that the experiments are impressive and appear well performed. The reviewers have specific suggestions for improving the manuscript related to data analysis, interpretation and discussion. Please respond to the comments of each reviewer as detailed below and make appropriate revisions to your manuscript.

Reviewer #1

In this manuscript, the authors examine the interaction between ‘rapid sniffing’ in awake mice and concentration-invariant coding of odor identity, using a combination of whole-cell recordings and behavioral assays. They find that rapid sniffing has a similar impact as increasing odor concentration on mitral and tufted cell responses, although in separate behavioral assays, sniffing behavior does not correlate with discrimination of different-concentration odors. They then provide evidence that this apparent confound can be explained by decoding sniff parameters from respiration-patterned activity across a population of mitral and tufted cells. While I have some reservations about the strength of their proposed model, it is an important idea and the study, on the whole, involves an extremely impressive set of experiments and contains a wealth of interesting data which are important for driving further exploration of these ideas. I congratulate the authors on this impressive work. There are a few important points regarding analysis and interpretation points that should be addressed, however, and discussion of these results in the context of prior studies could be improved, as detailed below.

We would like to thank the reviewers for their time and effort in reviewing the manuscript and for the helpful suggestions, and we are pleased that they find the study of potential importance to the field. We have addressed the important points raised with new data analysis, and modelling and additional / rewritten text in the revised manuscript.

Major points

1. One technical concern is that, as far I understand it, all analyses comparing concentration and sniff speed effects on firing rate and latency come from averages across multiple trials. The number of trials used for this averaging are not clear. For example, for the data shown and discussed in Figure 1C, D and E, from how many trials are firing rates averaged, for each cell? Likewise, for the latency data shown in Figure 2 and related text, how many responses were averaged to generate FR profiles in order to calculate response latency? This is important because of the possibility that some of the apparent effects of fast sniffing could result solely from averaging trials - for example, an increased reliability (or decreased baseline variance) from trial to trial during high frequency sniffing would be expected to generate stronger and faster average responses even when such an effect is absent on a single-trial basis. If there were a reasonable way to assess the impacts of fast and slow sniffing, and high and low concentrations, on a trial-by-trial basis (which is the situation the animal is faced with in reality) this would strengthen the overall conclusions.

Thank you for this important comment. We apologise for the lack of clarity in terms of trial number. In every comparison, we have made sure to keep trial numbers the same for the three conditions: low concentration + slow sniffing, high concentration + slow sniffing and low concentration + fast sniffing. Either we took a fixed number of trials in each case (e.g. five trials, as in Figure 1) or we took trials based on whether inhalation duration for the trial was within the upper of lower percentile (as in Figure 2) -- see methods section on lines 201-207 of the revised MS.

To clarify this, we have added the following text:

Lines 202 to 203: “Note that when comparing response changes due to concentration and response changes due to sniff change, the same number of trials was used for both conditions.”

Lines 358-362: “To compare changes in response for higher concentration to those for faster sniffing, we took trials from each stimulus concentration based on inhalation duration: slow sniffing trials (for both high and low concentrations) were taken as the five trials with highest mean inhalation duration (MID), and fast sniffing trials were the five trials with the lowest MID.”

We have now also demonstrated the reduction in response latency for faster sniffs on a trial by trial basis for two example cells in figure R1, from which panels A and B have been added to Extended data Figure 2-1F in the revised manuscript.

Figure R1. Spike responses across trials sorted by inhalation duration (now part of Figure 2-1)

(A) Left: plot to show first inhalation duration during odor stimulation sorted from smallest to longest for all trials for one cell. Middle: heatmap of spike count for the same cell during odor stimulation for trials sorted by first inhalation duration as in left plot. Right: Example spike counts for individual trials as marked by their asterisks in the middle plot. (B) As for A, but for a different example cell. Note the consistent reduction in latency of the spike response with decreased inhalation duration (faster sniffing) for both cells (the latency ‘saturates’ in the first example cell in A).

2. The odor concentrations used are, first, both quite high (in this reviewer's opinion) and, second, not all that different from one another: a 2.5-fold change in concentration (which is only 0.4 log unit change in intensity) does not present a particularly challenging problem in terms of concentration-invariant odor perception. This is relevant for alternate interpretations of the authors' results, as detailed in comment 3 below. Do the authors suspect that sniff speed mimics changes in odor concentration only over this modest range? Over what range do odor concentrations vary in a naturalistic plume? Addressing these questions in their Discussion would be helpful for thinking about the relevance of their model for behavior under naturalistic conditions.

This is indeed an important question. The stimulus concentrations were chosen initially for a practical reason: ‘high’ concentrations were used in order that a large number of cells would respond, enabling whole cell recordings from a relatively large portion of responsive cells. We deliberately chose small concentration differences as we expected sniff-related changes in representation to have the strongest effect on these fine discriminations. Given this, we find it surprising that mice can easily distinguish the small concentration differences (as shown in Figure 3 and 4) -- even in the first few trials of the task (Figure 3F), and yet sniff changes do not affect the decision of the animal in an overt way even for this very fine concentration discrimination (Figure 4).

The question of over what range sniff speed mimics concentration changes is difficult to answer without direct measurement of naris odor concentration, but we can make some tentative hypotheses. Fluid dynamic models predict that a sniff with a higher flow rate will cause the temporal profile of concentration in the naris to become steeper -- i.e. more odor molecules are drawn in per unit time (Shusterman et al., 2017; see bioRxiv, doi: 10.1101/174417). Thus we would expect the concentration change that a sniff change can mimic to be proportional to the environmental concentration. i.e, if the environmental concentration is 0.1 %, and sniff flow rate can change over a twofold range (previous measurements have shown that nasal airflow can vary over at least this range -- e.g. Youngentob et al., 1987), we would expect that sniff changes could mimic concentration differences in the range 0.1 to 0.2 % If the environmental concentration were 10 %, sniff changes could mimic concentration changes in the 10 to 20 % range. Indeed, for a large enough concentration difference, e.g. 0.1 vs 10 %, sniff changes will not completely obscure the difference, just make it slightly smaller.

Within natural plumes, odours come in packets and concentration can vary from a source-dependent maximal concentration to 0, the total absence of an odour. However, it is not currently known what the behaviourally relevant concentration information is in terms of e.g. odour source localisation -- i.e. whether average concentration is used, or whether the frequency of high concentration ‘spikes’ is used. It was clear from our behavioural task however that our mice could distinguish the CS- from CS+ concentration within the first 10 trials of the task (Figure 3F), and thus this did not require extensive training beyond the natural capability of the mouse.

We have added the following discussion on this topic to page 36:

“Here we chose relatively high concentrations (1-3% saturated vapor pressure) in order to ensure a good rate of response in whole cell recordings, and relatively modest concentration differences since we expected sniffrelated differences in representation to have the most pronounced effect on performance in these fine discriminations. The question then arises as to what concentration range sniff variations will impact concentration estimation. This is difficult to answer without direct measurement of naris odor concentration, but we can make some tentative hypotheses. Fluid dynamic models predict that a sniff with a higher flow rate will cause the temporal profile of concentration in the naris to become steeper -- i.e. more odor molecules are drawn in per unit time (Shusterman et al., 2017). Thus we would expect the concentration change that a sniff change can mimic to be proportional to both the environmental concentration, and the range over which nasal flow can change (previous measurements show that this is at least twofold -- e.g. Youngentob et al., 1987). ”

3. The behavioral data presented in the middle section are very nice and convincingly suggest that sniff speed (specifically, inhalation duration) is not a confound in discriminating different-concentrations. These results are used as the justification to search for mechanisms by which the ‘paradox’ of sniff-dependent changes in mitral/tufted cell responses is resolved - namely, the ‘reading’ of sniff parameters from a population of sniffdriven mitral/tufted cells, which is explored in the final section. There is nothing wrong with this idea, but to this reviewer there is a simpler explanation, which is that the mitral/tufted cell population code discriminating the different learned concentrations is sufficiently robust that it is not perturbed by the rather modest changes in firing rate or small changes in latency that accompany differences in sniffing. This could especially be true after the extensive training involved here, and if one considers the large numbers of neurons (far more than are recorded by the experimenters) that are available to contribute to robust odor identity coding. In short, the

‘paradox’ the authors nicely explain away may not be much of a paradox at all.

I do not mean to invalidate the final section of the study, which is quite nice in its own right, but this alternative explanation should at least be acknowledged.

This is indeed a possibility and an important point. Our results show that for latency or FR on the single cell level, fast sniffing at low concentration looks very similar to slow sniffing at high concentration. We would predict, for such latency or FR codes, that even a large population of such cells would not help distinguish the two. It is possible however that there is a small and specialised MTC subtype, or a population level code (ensemble code or sequence code) that is more robust in the face of sniff change that we cannot see with single cell recording. In either case, we do not think that the training mice undergo in our experiments would play a major factor, given that mice easily and rapidly identify the novel concentration as indicated by sniffing behaviour the first time it is presented. The training to criterion in this situation is likely to be conditioning of the response, rather than how to perceive the difference in concentration.

To clarify and discuss this important point we have added the following remarks to page 39-40:

“It is possible however that there is a small and specialised MTC subtype that might encode odor concentration in a simple, sniff-invariant manner. We deem this less likely since the accompanying study records from a much larger portion of MTCs, and finds that sniff variance still renders concentrations very difficult to discriminate (Shusterman et al., 2017). Alternatively, population level codes could be employed for concentration encoding (e.g. via spike synchrony or higher order features) which are robust in the face of sniff change but elude identification with single cell recordings. It is also possible that sniff-invariant features appear in the OB after training on the concentration task, however it must be noted that, in our hands, our mice could detect even the relatively small difference in concentration within the first presentation of the novel stimulus (Figure 4F), suggesting that fine odor discrimination occurs readily in a sniff-independent manner not requiring any extensive training.”

We have also reduced the strength of wording in our claims about the paradox and our proposed solution throughout the text.

4. There are a number of points in this paper which were addressed also in a recent publication from DiazQuesada et al. (J Neurosci 2018), yet this paper is not mentioned. It would be useful to note which results are similar to those reported in that study, and which are different. For example, the Diaz-Quesada study reported mixed effects of concentration on mitral or tufted cell response latency or amplitude, and a lack of correlation between effects of sniff speed and mitral versus tufted cell subtypes. Could these differences be related to recordings performed in awake versus anesthetized mice? Are there other explanations?

Thank you for drawing this omission to our attention. Indeed two of the major differences between our work and Diaz-Quesada et al. are 1) that anaesthetics (pentobarbital) are used and 2) that the study largely focusses on sniff frequency, and as such in the tracheotomised part of the study only this parameter is altered, while in awake mice several sniff parameters covary (see extended data Figure 1-2). As such, Diaz-Quesada et al do not study the effect of sniff speed per se (this would require that inhalation duration and flow rate to be altered), but rather the sampling rate, while in our study, the flow dynamics of each sniff are different. As such, we would not expect to see any such changes in response latency caused by sniff changes (as shown in figure 2 of our manuscript) in their study, as the flow dynamics for the first sniff are always the same (e.g. in Figure 4A of DiazQuesada, the early transient shows no difference). As such, their study focusses more on the effects of sustained changes in sniff rates on spike rates, for which they find little difference between superficial and deep cells (likely corresponding to TCs and MCs). Here, we do not report differences for the cell types in these metrics, since fast sniffing is not sustained for many seconds in our study. As for the mixed effects of concentration reported in their study, it is unclear why they do not see the relatively consistent reductions in latency for higher concentrations that have been reported in the literature for OSNs, MTCs and pyramidal neurons in piriform cortex (Ghatpande and Reisert, 2011; Rospars et al., 2000; Cang and Isaacson, 2003; Fukunaga et al., 2012; Sirotin et al., 2015; Bolding and Franks, 2017; Shusterman et al., 2017). This may be an effect of the pentobarbital, but this is merely speculation.

In all, we feel the differences in the experimental questions, experimental design and analyses make it difficult for meaningful comparisons between our study and Diaz-Quesada et al., however we now cite this otherwise highly relevant and interesting article in the introduction on lines 61-62.

Minor comments.

5. The Results relating to differential effects of rapid sniffing on putative mitral or tufted cells are confusing and somewhat circular. First, the epoch used to determine phase preference for cell type classification (line 395) is not stated; was this during odor presentation or during baseline respiration of air? More importantly, the fact that pTCs show less reduction in latency with fast sniffing than do pMCs is not surprising, since pTC responses are already at a minimal latency by definition (this is why they are labelled pTCs). This should be acknowledged.

We apologise for this omission and resulting confusion. Putative classification of MC and TC was based on sniff cycle phase preferences of membrane potential during baseline respiration of air, and the phases used to separate the two groups was based on previous work (Jordan et al., 2018; Fukunaga et al., 2012). Thus this is not a circular analysis, but cell type identification was based on epochs in absence of odor, and latency analysis conducted during the odor epoch.

We have now added this detail to the main text in line 413, and have expanded the “Phase preference and putative MC and TC boundaries” section in the methods.

6. Details of how ‘nasal flow’ was recorded are missing. What sensor was used? Was this recorded external to or internal to the naris? Was this an actual flow sensor or simply a pressure sensor in which flow is inferred? These details are relevant to understanding how sniff parameters are derived for their analyses.

We sincerely apologise for this omission, we have added a ‘Sniff Measurement’ section to the Methods on page 8 to describe this.

7. In Extended Data 1-2 (correlation of different sniff parameters), panel C appears to show that, at least in this animal, at sniff durations above approx 250 msec (4 Hz sniff frequency), there is no relationship between sniff duration and inhalation duration. For durations below 250 msec, a significant correlation is expected due to constraints of duty cycle for inspiration and exhalation. Thus, in my opinion the ‘significant’ correlation

reported here is not informative.

This is an important point. Indeed there is a plateau in the relationship below sniff frequencies of 4 Hz. We have now made linear regressions separately for the two phases of the relationship: 1) below 250 ms sniff cycle durations, and 2) above 250 ms sniff cycle durations (Figure R2). We have replaced Extended data Figure 1-2C with panel A and C from Figure R2.

8. The following text, as written, does not make sense: “Sniff cycles from 42 recorded neurons (25 MC, 17 TC) were divided into fast (37-80 ms), medium (80-96 ms) and slow (96-183 ms) cohorts. All cycles with sniff duration below the 0.5th percentile (108 ms) and above the 99.5th percentile (597 ms) were discarded. ” I believe the authors are referring to inhalation duration in the first sentence when defining fast, medium and slow cohorts, not sniff cycle duration as is implied. Please clarify.

Thank you for pointing out this error -- the numbers indeed refer to inhalation durations. This has now been corrected.

Figure R2. Relationship between inhalation duration and sniff duration. (Part of Extended data Figure 1-2)

(A) Scatter plot between sniff duration and inhalation duration for an example nasal flow recording. Linear regression models were produced 1) for all sniff cycles of duration &lt; 250 ms (magenta plot), and 2) for all sniff cycles &gt;250 ms (blue plot). (B) Linear regression models (as in A) from all 45 whole cell recordings. Thick plots show mean model across all recordings. (C) Histogram of R values for all linear regressions across the 50 recordings, top: for linear regressions made for sniff cycles &lt; 250 ms duration and bottom: for regressions made for sniff cycles &gt; 250 ms.

Reviewer #2

The paper entitled “Sniffing fast....” explores the inter-relationships between concentration and sniff rate, and asks about their relative effects on both MT cell firing and concentration discrimination in awake mice. The paper finds that changes in sniff rate mimic the effects of concentration changes on firing rates and dynamics (although not necessarily at the subthreshold level), but that the brain could potentially take advantage of the broad changes in firing rates apparent in odor-non-responsive neurons to know whether a given sniff was fast or slow, and thereby marginalize out the temporal effects of sniffing to enable concentration-invariant odor identity encoding. This paper is very carefully done, the similarities between changes in concentration and sniffing are eerie (and therefore provocative) and the analyses are convincing - it will be an important contribution to the field, and it offers an interesting feed-forward model for the conceptual problem of stimulus invariance. I have only a few minor comments regarding the manuscript:

Thank you for your encouragement and the helpful suggestions that we have addressed with new analysis and substantial additional modelling in the revised manuscript.

1. Many of the conclusions rest upon figure 2G, in particular those about ‘discriminability.’ However no direct analyses of discriminability (from a classification or an information-theoretic perspective) are actually performed. Given the data in the figure this should trivially work if there is enough data, and I'm assuming this type of analysis was not included because of the relatively small number of cell-odor pairs included in the analysis, but if possible some additional formalization of this claim would be helpful. This is not critical to the paper - just a suggestion.

This would indeed be the ideal analysis, however unfortunately due to the relatively small quantity and short duration of whole cell recordings, the number of trials for each condition is limited. However, the question of discriminability is dealt with in much depth by an accompanying paper using unit recording, already available on bioRxiv -- doi: 10.1101/174417 -- Shusterman et al., 2017).

Since there is little we can do analytically at this point, we have adjusted our discussion about discriminability based on the Euclidean distance analysis on page 23 of the revised manuscript.

2. Similarly, with the decoding network in figure 5, it is not obvious which neurons you actually need to do the decoding, nor is it really clear whether the best you can do is binary inhalation speed classification, an important point since the distribution of sniff speeds isn't bimodal. Some simple playing with off the shelf SVMs here could strengthen this argument by allowing better fractionation of sniff rates (and maybe identify specific cells that are particularly good at carrying this kind of information). Given that you are looking at odor-non-responsive cells here, you might have enough data for this sort of analysis but, again, if you don't have enough data this is just meant as a suggestion.

Thank you for this important suggestion. We have now generated a linear model to predict inhalation durations with a resolution of 10 ms based on peak firing rates of 25 cells with a sufficient number of sniffs in each time bin. The model was generated using 13 sniff cycles of a given inhalation duration, and tested on 7 sniff cycles (Figure R3A). Considering that this dataset is relatively limited in terms of cell number and trial number (particularly for certain inhalation durations), this classifier performs very well (correlation between actual and predicted inhalation duration, Pearsons r = 0.79; Figure R3B). When we compare the weights (regression coefficients) for the different cells in the model, we find that the large majority of cells are involved in the classification (Figure R3C), but coefficients for pMCs tend to be stronger in absolute terms than for pTCs and show both positive as well as negative values, while pTCs are weaker and almost exclusively positive (this results in significantly larger variance for pMC weights: p = 0.01, Bartlett test, Figure R3C). The weights are correlated with the degree to which peak firing rates were correlated with inhalation duration (correlation between log10(absolute regression coefficient) and the R2 value between inhalation duration and average peak firing rate:

R2 = 0.26, p = 0.01; Figure R3D), confirming that cells which were found to have stronger correlations as in figure 5, are also the most informative in the model.

Figure R3. Linear model classification of inhalation durations using peak firing fates (Part of figures 5 and 5-2 in revised MS)

(A) Histogram to show number of sniff cycles within each inhalation duration time bin used to generate the linear model (black) and subsequently test the linear model prediction (green). (B) Scatter plot between inhalation duration predicted by the model, and true inhalation duration for all 7 sniff cycles tested in each category. (C) Bar graph to show resulting weights (regression coefficients) assigned to each cell after in the linear model, sorted by weight for putative MCs and putative TCs. Boxplots show distributions for pTCs and pMCs separately. (D) Correlation between -log10(absolute regression coefficient) and the R2 value between inhalation duration and average peak firing rate.

We now include Figure R3B in place of Figure 5E (previous Figure 5D is now in Extended data Figure 5-2A), and Figure R3C to Extended data Figure 5-2C. This new analysis is discussed on pages 33-34 of the results of the revised manuscript.

3. The one conceptual concern here is that there are small populations of glomerulus-attached MT cells that, because they are connected to high-affinity receptors for a particular odor, operate in a quantitatively different regime than the remainder of the MT cells in the bulb. My superficial read of the recent Nature Comms Rinberg et al paper, which uses the high-affinity ligand for M72, suggests that these neurons might fire sooner than the distribution reported here for slow sniffs; it is formally possible, then, that these neurons could be report concentration changes on the low end in a manner that is invariant to sniff changes. The paper name-checks this idea in the discussion by saying that you might need to look at more neurons to be really sure that you aren't missing anything, but a couple more sentences about this specific possibility would make this caveat more clear (and wouldnot really take anything away from the paper - the fact that you can decode sniff rate from non-responsive neurons is incredibly important whether or not there is the ability to discriminate concentrations at the low end built into the system, and of course this high-affinity mechanism wouldn't apply to high concentrations, where perceptual invariance still applies and is a problem).

Thank you for raising this important point. There is certainly a possibility that there is a subpopulation of cells (or an ensemble timing code invisible in single cell recordings) capable of robustly coding concentration differences in the face of sniff changes. However, we consider this less likely for two reasons, both addressed in a co-submitted paper that is available on BioRxiv (Shusterman et al., 2017, doi: 10.1101/174417): 1) The mechanism for the reduced latency of response for both faster nasal flow and increased concentration is thought to be similar -- in both cases the concentration profile in the naris is steeper, and OSNs depolarise to threshold more rapidly. If this is indeed the case, information about the sniff (either nasal pressure or efference copy) must be integrated to distinguish the two scenarios. It remains possible that a small subpopulation in the OB is capable of this comparison. However, 2) the aforementioned study records from a larger population of cells extracellularly, and this theoretical subpopulation does not become apparent when attempting to discriminate concentrations. With regard to the possibility that the MTCs connected to the most sensitive glomeruli are capable of robustly encoding the concentration change, we consider this unlikely due to point 1: i.e. even at the low end of the concentration range, where these ‘first responders’ are presumably not saturated in terms of FR or latency, a concentration increase is expected to reduce latency and increase FR, while the same would also be expected for a sniff of higher flow rate, since the concentration profile in the naris is steeper.

We now explicitly discuss these ideas, in particular the recent evidence from Rinberg and colleagues. We have elaborated on this in the discussion as follows (page 39 of revised MS):

“Our results show that for response latency or FR on the single cell level, fast sniffing at low concentration looks very similar to slow sniffing at high concentration. We hypothesise that the mechanism for the reduced latency of response for both increased nasal flow and increased concentration is similar -- in both cases the concentration profile in the naris is steeper, and OSNs depolarise to threshold more rapidly. At the lower end of the concentration scale, this would even occur for the most highly sensitive ‘first responding’ MTCs that have been hypothesised to account for sniff and concentration invariant odor identity codes (Wilson et al., 2017).”

4. The legend to 5B threw me - because 5B2 includes the phrase ‘per sniff’ but 5B1 does not I though these were different (and strange) analyses of the same data, rather than of two different cells.

Thank you for highlighting this, this has now been corrected.
