# Supplementary figures and images for: Sniffing Fast: Paradoxical Effects on Odor Concentration Discrimination at the Levels of Olfactory Bulb Output and Behavior
Source: eNeuro. 2018 Dec 26;5(5):ENEURO.0148-18.2018. doi: 10.1523/ENEURO.0148-18.2018 (PMC6306510; doi:10.1523/ENEURO.0148-18.2018)

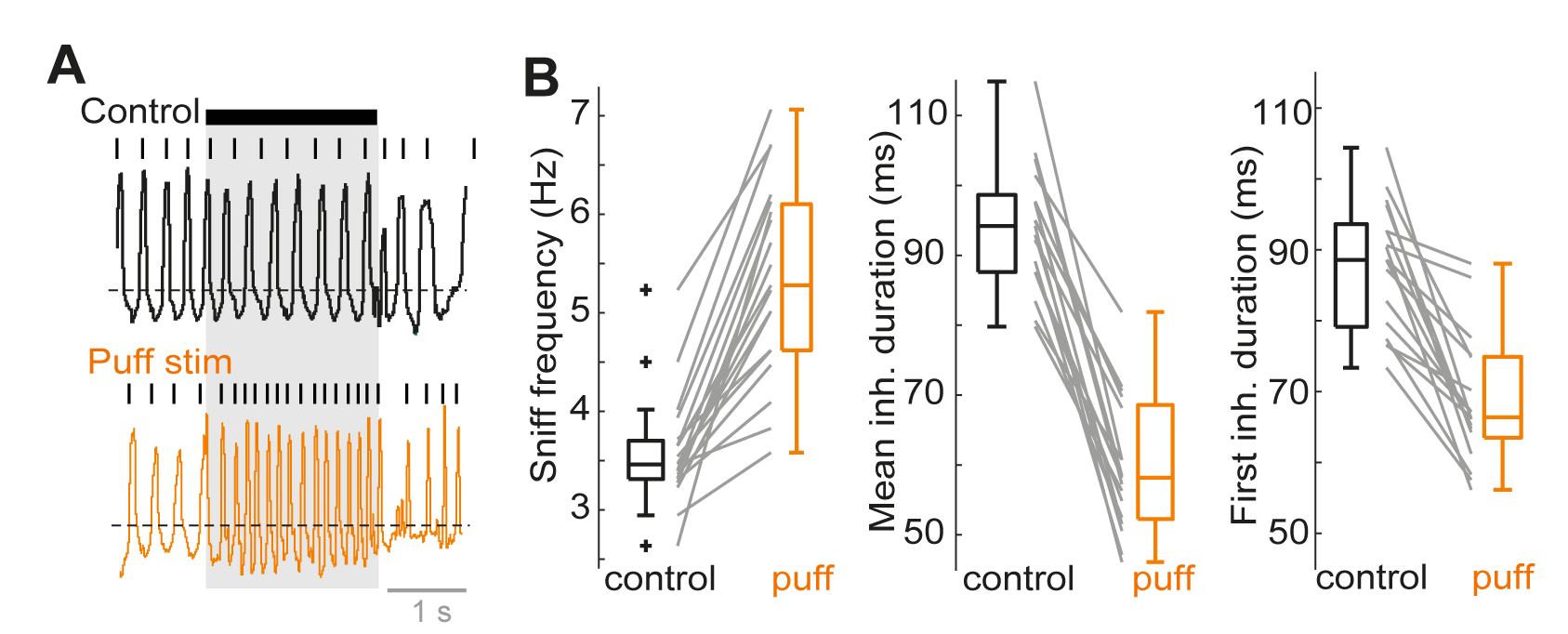

Supplement: Extended Figure 1-1 — Effect of puff stimulus on sniff behaviour. (A) Example nasal flow traces from one animal during a control trial (no puff stimulus accompanying 2 s odor stimulus) and a trial with a puff stimulus. Shaded area shows odor stimulus. Black ticks indicate inhalation onsets. (B) Plots to show average change in sniff frequency, mean inhalation duration, and first inhalation duration between five control (black) and five puff (orange) trials for all 20 cell-odor pairs. Download Extended Figure 1-1, TIF file. [file sup_enu-eN-NWR-0148-18-s08.tif]

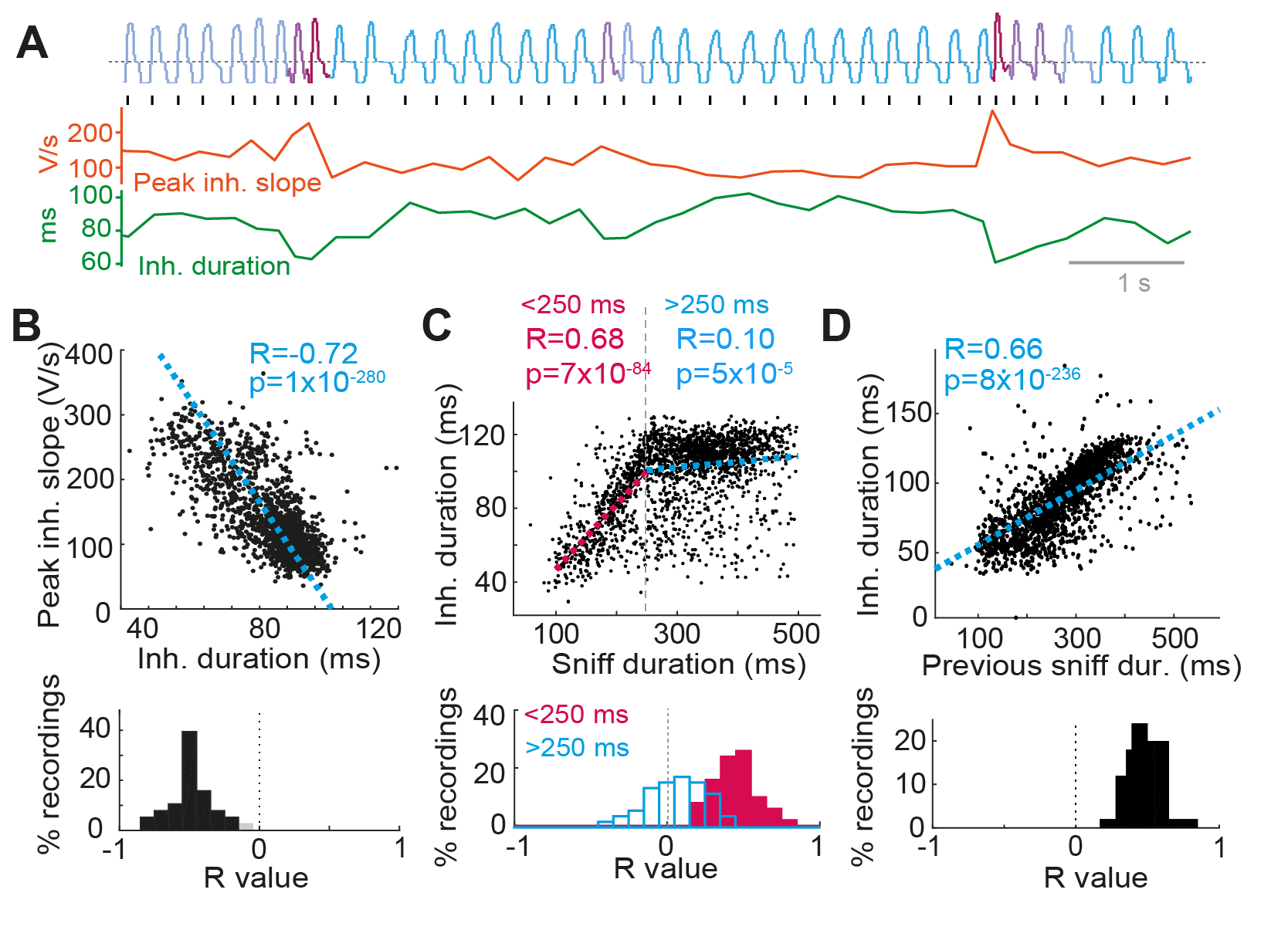

Supplement: Extended Figure 1-2 — Relationships between different sniffing parameters. (A) Example nasal flow trace during an inter-trial interval (no odor), with sniffs colored according to their inhalation duration (blue to red = long to short duration). Black ticks show time of inhalation onset, orange plot shows peak inhalation slope for each inhalation aligned to the inhalation onset, and green plot shows inhalation duration for each inhalation. (B) Example correlation between inhalation duration and peak inhalation slope for 1988 sniffs in 1 animal (top), and histogram of correlation R values between inhalation duration and peak inhalation slope across 50 animals. Black bars indicate significant correlations. (C) As for B, but for correlation between sniff duration and inhalation duration. As expected from constraints on duty cycle during respiration, we found a biphasic relationship between sniff duration and inhalation duration, with a linear correlation for sniffs < 250 ms duration (magenta) and a plateau for sniffs > 250 ms duration (blue). This resulted in high R values for sniffs < 250 ms duration and low R values for sniffs > 250 ms duration. (D) As for B, but for the correlation between the previous sniff duration and the current inhalation duration. Download Extended Figure 1-2, TIF file. [file sup_enu-eN-NWR-0148-18-s07.tif]

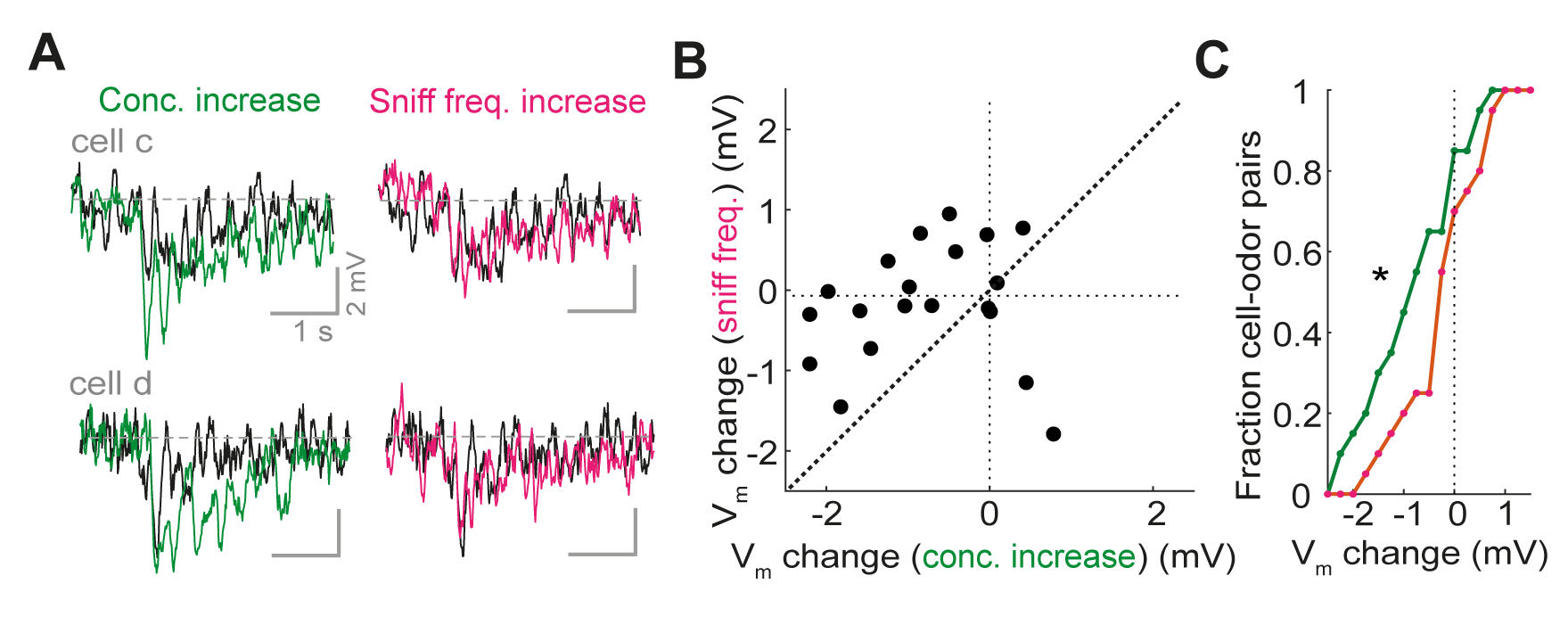

Supplement: Extended Figure 1-3 — Changes in subthreshold response are more inhibitory for concentration increase than for fast sniffing. (A) Example average subthreshold response traces for low concentration, slow sniffing (black), high concentration, slow sniffing (green) and low concentration, fast sniffing (magenta), for two different cells, cell c (top) and cell d (bottom). Each trace is the average of 5 spike-subtracted trials. (B) Scatter plot to show average change in membrane potential response for the first 1 s of the odor stimulus for concentration increase (high conc.-low conc.) and sniff frequency change (fast sniffing-slow sniffing). (C) Cumulative histograms of membrane potential response change for concentration increase (green) and sniff frequency increase (magenta). P = 0.03, paired t-test. Download Extended Figure 1-3, TIF file. [file sup_enu-eN-NWR-0148-18-s06.tif]

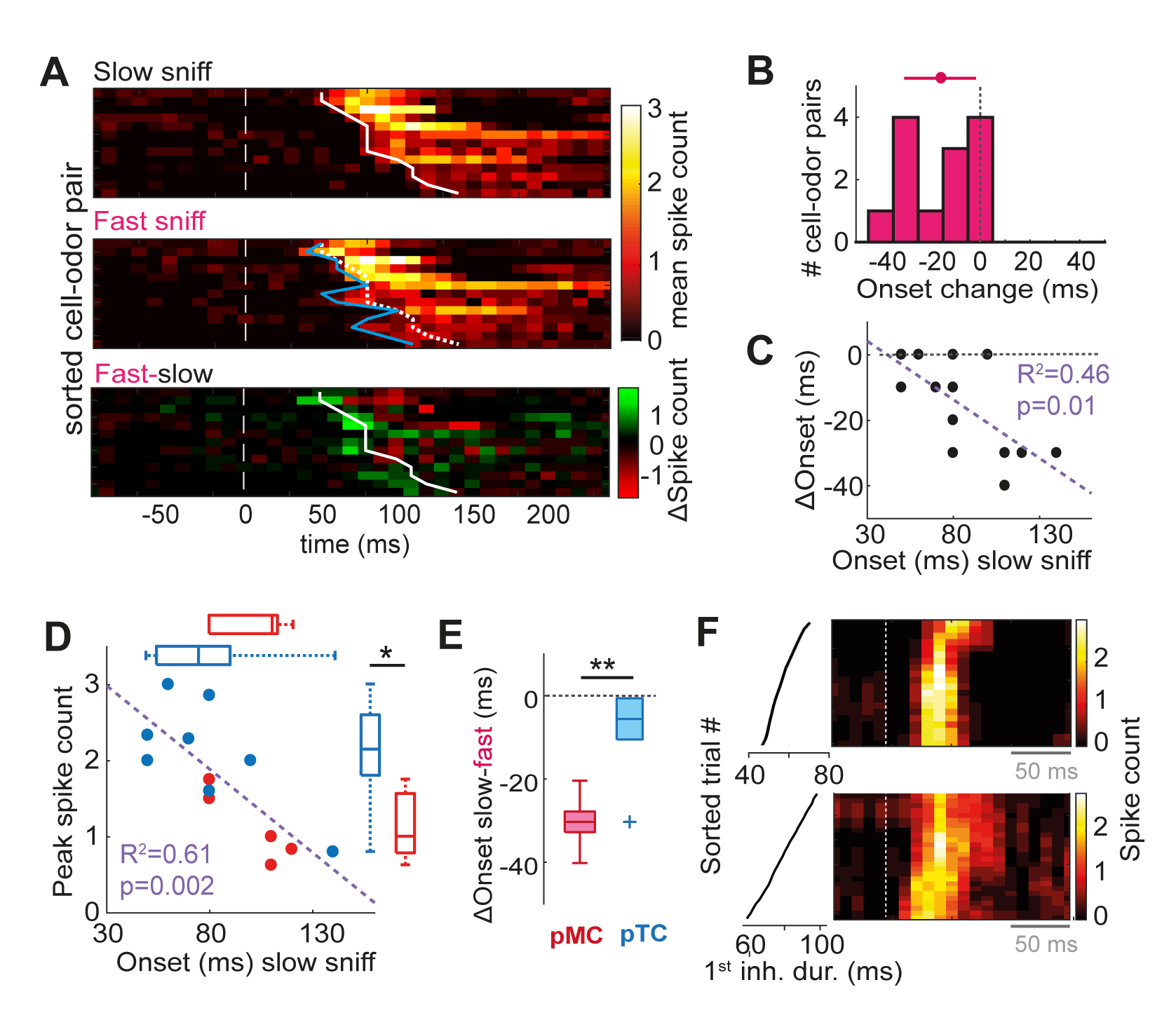

Supplement: Extended Figure 2-1 — Additional data for sniff-induced temporal shifts in odor response. (A) Heatmaps of mean spike count for 13 cell-odor pairs showing early excitation in response to the odor presented, for both slow inhalation (top) and fast inhalation (middle). White dashed line indicates odor onset aligned to the first inhalation onset. Cell-odor pairs are sorted from short to long response onset latency (during slow inhalation). Bottom heatmap shows the difference between the two above (fast-slow). White solid and dotted line indicates onset latency of each cell-odor pair for slow inhalation. Blue line indicates onset latency for fast inhalation. (B) Histogram of onset latency changes (fast-slow) for all 13 cell-odor pairs. Errorbar shows mean and SD. (C) Scatter plot to show relationship between onset latency for slow inhalation, and the onset change between fast and slow inhalation (ΔOnset). (D) Correlation between response onset latency and peak spike count (analysed within 10 ms time bins) for early excitatory odor responses evoked by a slow sniff. Blue data comes from pTCs and red data comes from pMCs. Boxplots compare the two parameters for pTCs and pMCs. (E) Comparison of response onset latency change (fast-slow sniff) for pMCs and pTCs. (F) Above and below plots are for two different example cells. Left: plot to show first inhalation duration during odor stimulation sorted from shortest to longest for all trials for one cell. Right: heatmap of spike count for the cell during odor stimulation for trials sorted by first inhalation duration as in left plot. White dotted line indicates where odor is on (aligned to first inhalation onset). Download Extended Figure 2-1, TIF file. [file sup_enu-eN-NWR-0148-18-s05.tif]

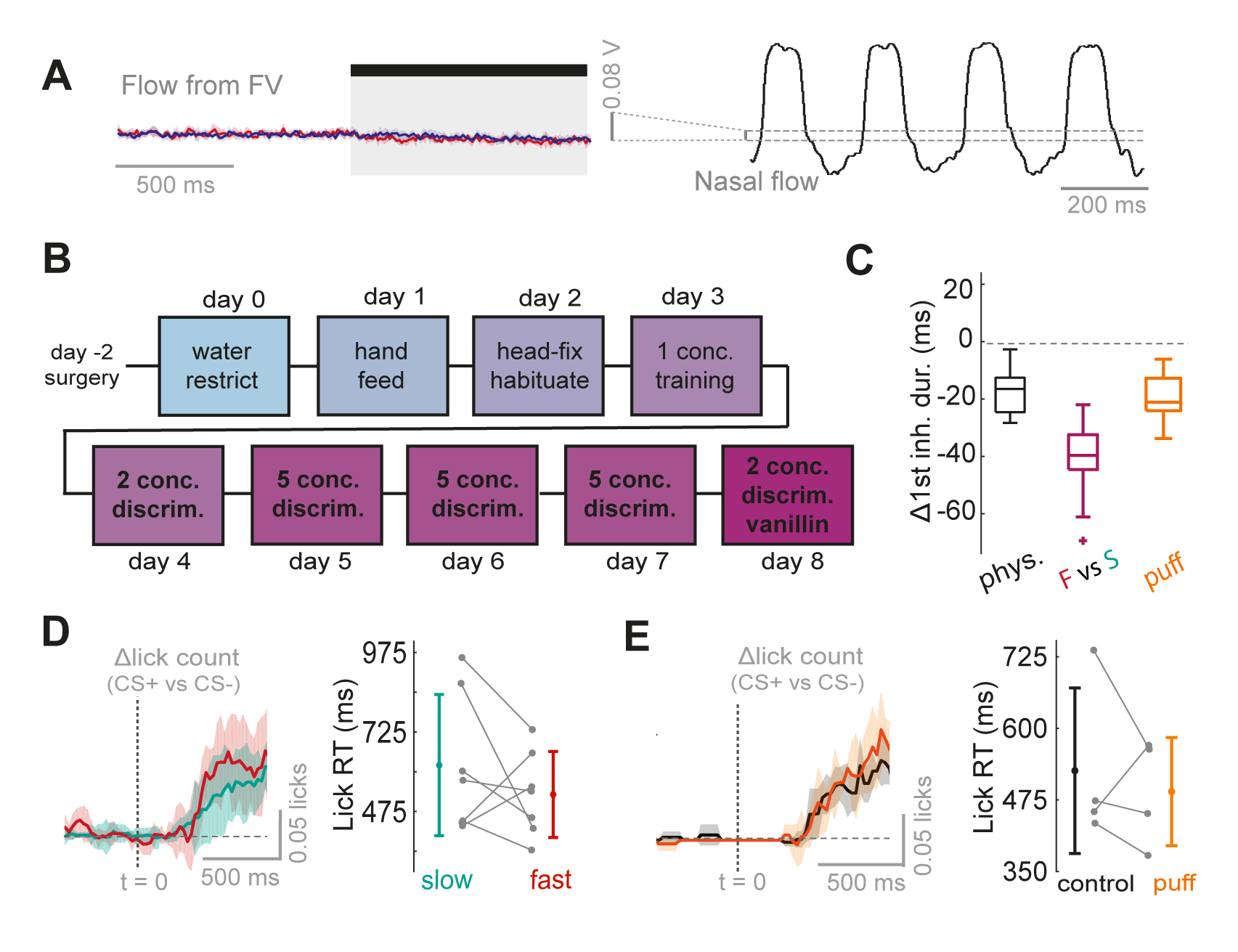

Supplement: Extended Figure 3-1 — Additional behavioral data. (A) Mean flow change recorded 1 mm from olfactometer output for high concentration stimulus (red) and low concentration stimulus (blue). Average of 10 trials; shaded area shows standard deviation. Y scale bar is compared to that of nasal flow traces recorded in the same manner to demonstrate the negligible nature of flow change from the olfactometer. (B) Diagram to show training sequence for mice (described in methods). (C) Comparison of changes in the first inhalation duration between physiological and behavioural experiments. Black ('phys.') shows distribution of mean change used for analysis of odor responses for 20 cell-odor pairs recorded in passive mice (as in Figure 2). Purple ('F vs S') shows mean difference between red and cyan sections of the inhalation distribution as in Figure 4F for all mice and concentrations (n = 7 mice x 5 concentrations). Orange 'puff' shows average changes in mean first inhalation duration for puff vs control trials during behaviour (n = 7 mice x 5 concentrations, as in Figure 4J). (D) Average difference in lick-histograms between CS+ and CS- (concentration 4, 2.6%, vs concentration 2, 1.4%) averaged across all 7 mice for slow sniff trials (cyan data) and fast sniff trials (red data) partitioned as in Figure 4F. Dotted line indicates onset of odor stimulus (aligned to the first sniff onset). Right plot shows difference in reaction times as measured by licking for fast and slow sniff trials for all 7 mice. Mean difference in RT (fast-slow) = -92 ± 235 ms, p = 0.34 paired t-test. (E) As for panel D, but now comparing lick distributions and reaction times between puff trials (orange) and control trials (black), as in Figure 4L. Mean difference in RT (puff-control) = -36 ± 125 ms, p = 0.61, paired t-test. Download Extended Figure 3-1, TIF file. [file sup_enu-eN-NWR-0148-18-s04.tif]

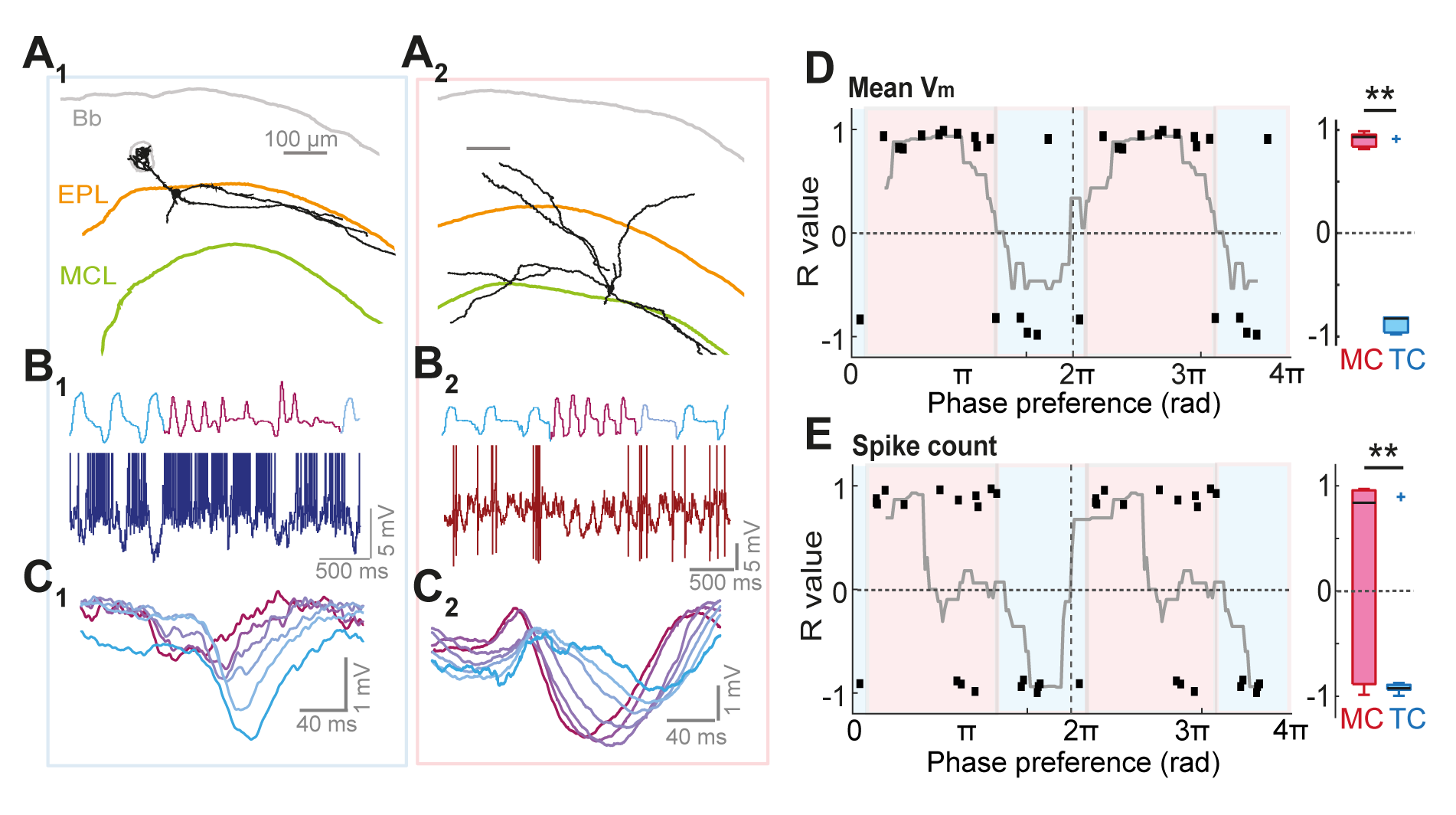

Supplement: Extended Figure 5-1 — Cell type specificity of effect of inhalation as defined by sniff-phase preference. In absence of applied odor, putative mitral cells (pMCs) respond to faster sniffs with increases in inhibition, and putative tufted cells (pTCs) with increases in excitation. (A1) Reconstructed morphology of a tufted cell recorded in awake mouse. 'Bb' refers to brain border, 'EPL' refers to external plexiform layer and 'MCL' refers to mitral cell layer (these morphologies have been previously published in Jordan et al. 2018 for different purposes). (B1) Example nasal flow and Vm trace during a rapid sniff bout (blue to purple represents longer to shorter inhalation duration on flow trace. Spikes have been cropped for display. (C1) Mean membrane potential waveform for different bands of inhalation duration: blue = long inhalation duration, purple = short. (A2)-(C2) as for A1-C1, but for a filled mitral cell recorded in an awake mouse. (D) R values for correlations between inhalation duration and mean Vm as a function of phase preference. Only strong correlations have been included (p<0.05 and R2>0.6). Grey line shows mean R value for all cells within a 2 radian moving window (centred), to give an idea of the phase modulation strength of the data. There was a significant organisation according to phase (p<0.01, bootstrapping, see methods). Boxplots to the right compare all values within the putative MC (red) and putative TC (blue) phase boundaries (mean Vm: pMC: median = 0.93, IQR = 0.84 to 0.95, n = 6; pTC: median = -0.83, IQR = -0.96 to -0.82, n = 10; p = 0.002, Ranksum). These phase boundaries are based upon those used in previous studies (Jordan et al., 2018; Fukunaga et al., 2012). (E) As for panel D, but for mean spike count per sniff. Again, there was a significant organisation according to phase preference (p<0.001; bootstrapping, see methods), and R values were significantly different between pMC and pTC boundaries (spike count: pMC: median = 0.84, IQR = -0.88 to 0.96, n = 22; [file sup_enu-eN-NWR-0148-18-s03.tif]

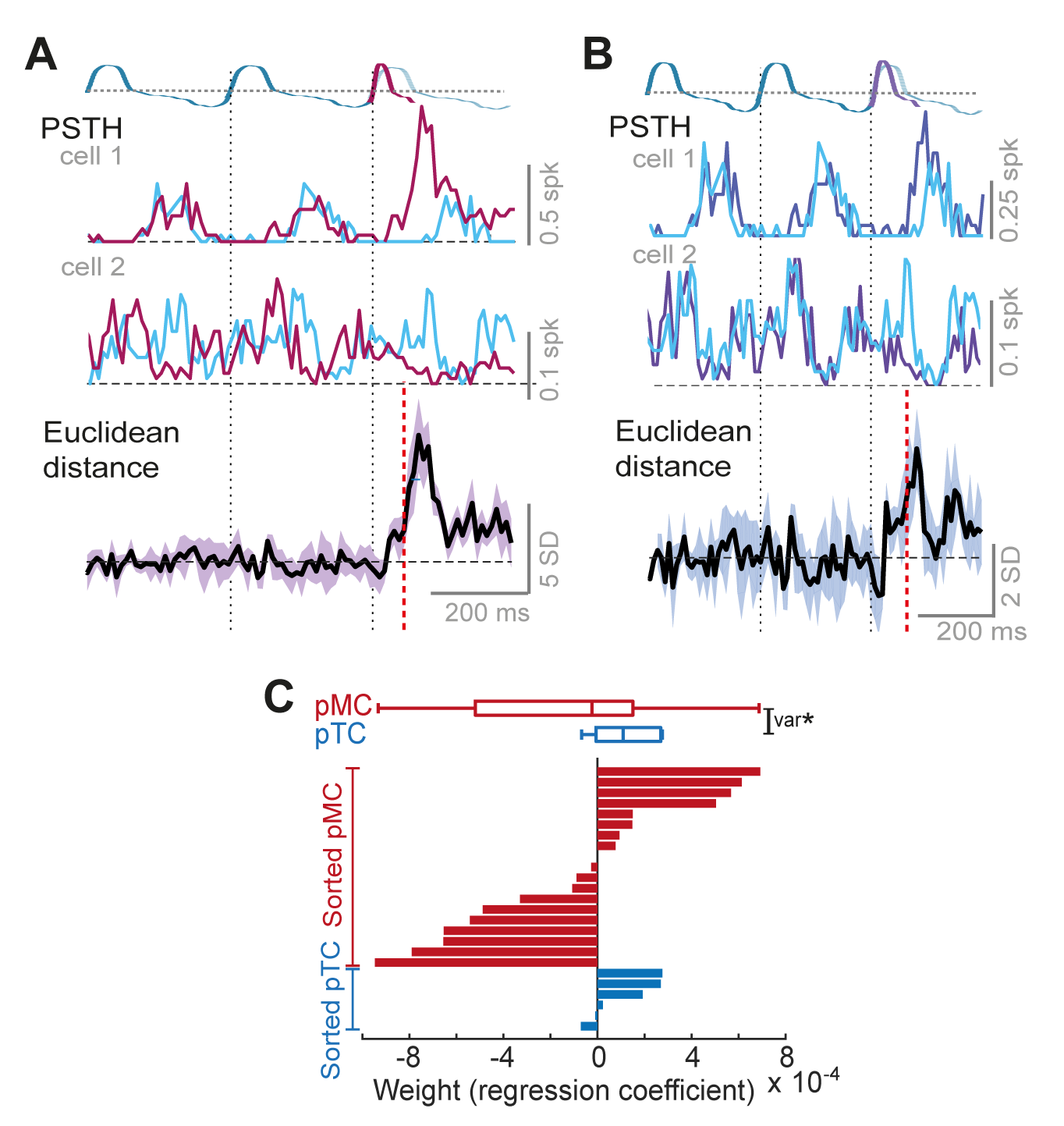

Supplement: Extended Figure 5-2 — Detection of inhalation duration change and cell 'weights' in linear model (A) Top: diagram to show construction of sniff sequences of different inhalation duration: either three of 95 ms inhalation duration (blue), or two of 95 ms with the final sniff of 55 ms duration (purple). Below shows two example PSTH sequences (from two different cells) averaged from random subsets of 25 sniffs that show the particular inhalation duration. Blue plot shows the PSTH sequence for 4 sniffs of 95 ms, and purple plot shows sequence in which the last inhalation is of 55 ms. Bottom trace shows mean Euclidean distance calculated between population vectors containing all cells constructed from the two sniff sequences as in panel A. Plot shows the average of 5 different subsets of data (made by averaging different sniff subsets for each cell), and shaded area shows standard deviation. Dashed red line indicates time of significant detection of change. (B) As for panel A, this time comparing PSTHs for a smaller inhalation duration change (95 ms, blue to 75 ms, purple). (C) Regression coefficients (weights) for individual MTCs in the linear model used to predict inhalation duration based on peak spike rates (related to Figure 5E; see methods). Download Extended Figure 5-2, TIF file. [file sup_enu-eN-NWR-0148-18-s02.tif]

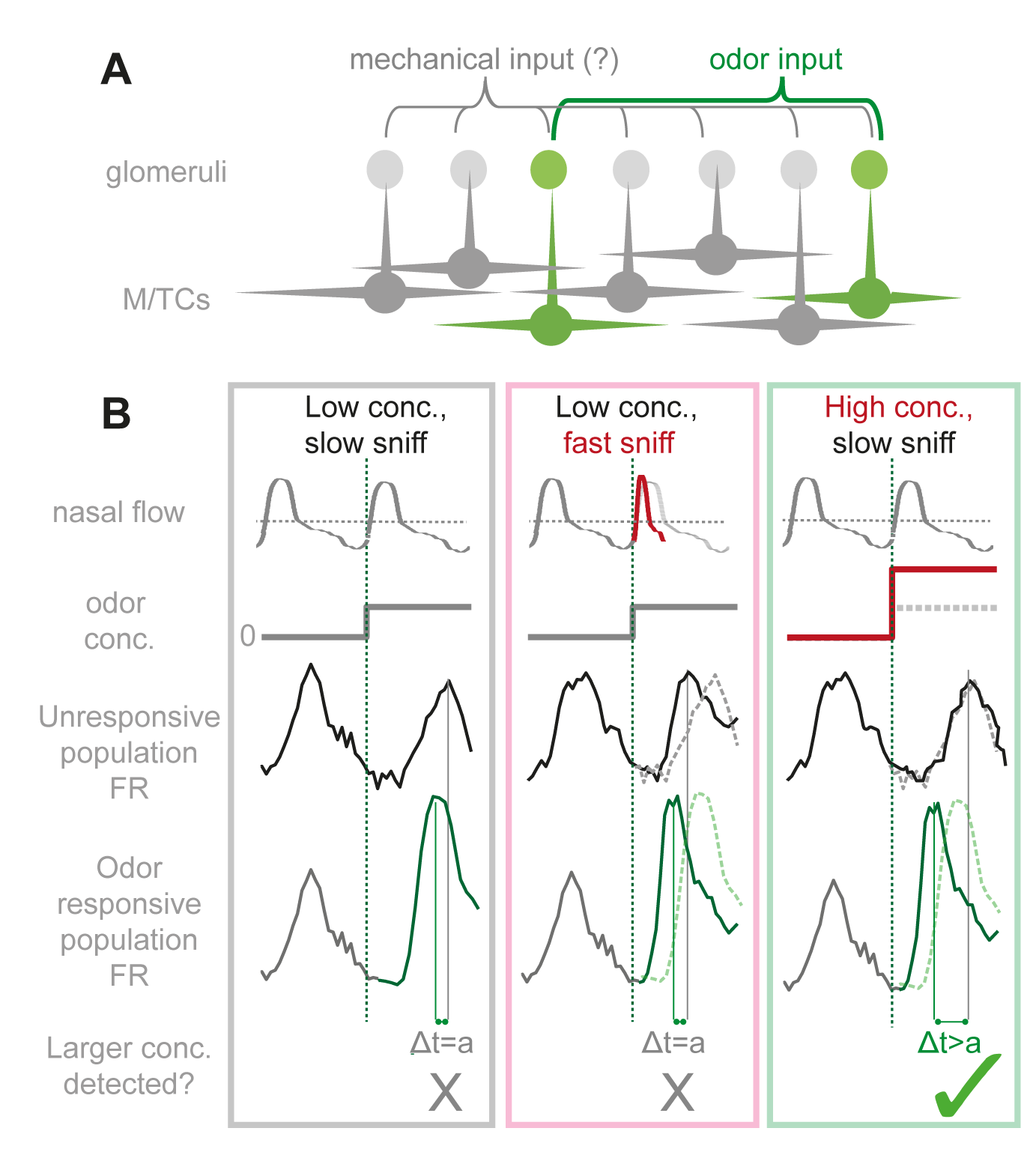

Supplement: Extended Figure 5-3 — Diagram of a potential relative timing code for concentration. (A) Highly simplified diagram of the olfactory bulb, depicting only glomeruli and MTCs. Green glomeruli/MTCs show those receiving odor input (odor responsive), while grey glomeruli/MTCs show those with absence of such input (unresponsive population). While odor inputs are sparse, mechanical input (response to the pressure change associated with each sniff) is widespread. (B) Diagram to show how a relative time code may work. Each instance shows one sniff prior to and during odor stimulation. 'Nasal flow' shows these two sniffs, with grey showing slow sniffs (>95 ms inhalation duration) and red showing a fast sniff (e.g. 55 ms inhalation duration). 'Odor concentration' trace shows a step increase in concentration from zero just prior to the second sniff. Grey traces show low concentration and red shows high concentration (2 x low concentration). 'Unresponsive population' shows a hypothetical population average FR for all cells without odor inputs during the odor stimulus. Dotted traces in second and third column for the second sniff cycle show the trace for the first column, for sake of comparison. 'Odor responsive' shows the average population FR for cells receiving direct odor input. Dotted traces in second and third column for the second sniff cycle show the trace for the first column, for sake of comparison. Δt shows the difference in peak population activity for unresponsive and odor-responsive cells during the stimulus. Note that Δt remains stable unless the concentration changes, and not when the sniff alone changes. A change in Δt allows perception of a different concentration. Download Extended Figure 503, TIF file. [file sup_enu-eN-NWR-0148-18-s01.tif]
